# A Scoping Review of the Conceptualization, Operationalization, and Institutional Recognition of the Scholarship of Teaching and Learning in Health Professions Education: Using Institutional Logics to Understand Inconsistencies

**DOI:** 10.5334/pme.2740

**Published:** 2026-06-05

**Authors:** Lara Varpio, Flynn Jones, Jennifer Lege-Matsuura, Katherine Schultz, Jonathan Amiel, Kim Dunleavy, Gail M. Jensen, Pamela R. Jeffries, Rachel Ellaway

**Affiliations:** 1 Department of Pediatrics, Perelman School of Medicine, University of Pennsylvania, USA; 2 Medical Education Collaboratory, The Children’s Hospital of Philadelphia, USA; 3 Holman Biotech Commons, University of Pennsylvania Libraries, USA; 4 Department of Pediatrics, University of New Mexico School of Medicine, USA; 5 Office of Professionalism & Inclusion in the Learning Environment, New York-Presbyterian, USA; 6 Department of Physical Therapy, College of Public Health and Health Professions, University of Florida, Gainesville, Florida, USA; 7 Department of Physical Therapy, School of Pharmacy and Health Professions, Creighton University, Omaha, NE, USA; 8 Vanderbilt School of Nursing, Nashville, Tennessee, USA; 9 Department of Community Health Sciences, Cumming School of Medicine, University of Calgary, Calgary, Alberta, Canada

## Abstract

**Introduction:**

The Scholarship of Teaching and Learning (SoTL) is the practice of critically examining student learning to improve teaching and then disseminating insights from this work for others to build on. SoTL often justifies faculty members’ careers in health professions education (HPE); however, SoTL is not universally understood or acknowledged in promotion and tenure (P&T) decisions across HPE institutions. We conducted a scoping review of the published literature to map how SoTL has been conceptualized, realized, and described as institutionally recognized by P&T committees.

**Methods:**

In July 2024 (updated in July 2025 and April 2026), the authors searched 7 databases for peer-reviewed publications referring to any scholarship relating to education, teaching, and/or learning in HPE in the United States. We followed Arksey & O’Malley’s scoping review methodology and incorporated stakeholder feedback. Articles were grouped by discipline: medicine and other health professions.

**Results:**

Of the 4,588 articles identified, 130 were included for full review, with 52 reporting on SoTL activities in medical education (40%) and 78 in other HPE domains (60%). While articles shared core SoTL concepts, SoTL was operationalized in a variety of ways, many blurring the distinction between scholarly teaching and SoTL. The articles demonstrated that SoTL has been inconsistently recognized by P&T committees.

**Conclusion:**

Using Institutional Logics to support analysis, we found that the recognition of SoTL in HPE organizations varies with the degree of alignment between the logics of research and teaching. We propose strategies for increasing this alignment and provide literature-based distinctions between scholarly teaching, SoTL and educational research to support this goal.

## Introduction

Defined as “a broad set of practices that engage teachers in looking closely and critically at student learning in order to improve their own courses and programs, and to share insights with other educators who can evaluate and build on their efforts” [1 (p. xix)], the Scholarship of Teaching and Learning (SoTL) is a foundational aspect of health professions education (HPE). The underpinning premises of SoTL—i.e., that the academic work of education can (and should) be recognized as scholarship when it is performed with rigor, and when it is disseminated to peers for evaluation and adoption in their practices—have grounded arguments justifying HPE as a field of inquiry [2,3,4]. They have underpinned calls for formally recognizing the academic activities of health professions educators in promotion and tenure (P&T) decisions [5,6,7,8]. And yet, while this has fostered impressive growth, some authors have warned that SoTL is not universally acknowledged in HPE, nor is it consistently rewarded by P&T committees as equivalent to research [9]. This incongruity was recently confirmed at a SoTL-focused workshop sponsored by the National Academies of Science, Engineering and Medicine’s Global Forum on Innovation in Health Professions Education. At this meeting, presenters reported that institutional support of SoTL is divided, with workshop participants (n = 25, representing 11 health professions) recounting that HPE institutions fall into one of two groups: organizations either strongly supported faculty members’ engagement in SoTL during P&T decisions or largely failed to acknowledge SoTL activities [10]. This suggests that the promise of SoTL in HPE has yet to be consistently realized.

Discussions of SoTL in HPE are often grounded in the work of Boyer [11], Glassick et al. [12], and Hutchings & Shulman [13]. In 1990, Boyer upended traditional ways of thinking about scholarly work by proposing that academics can legitimately engage in four distinct but overlapping forms of scholarship: discovery (i.e., basic research), application (i.e., applied research and engagement), integration (i.e., knowledge synthesis), and teaching (i.e., educational development) [11]. Boyer rejected the widespread positioning of the scholarship of discovery as the most valuable form of scholarship, arguing that all four approaches were equally worthy of recognition. While support for Boyer’s arguments spread quickly [14], his work did not include standards for evaluating this wider framing of scholarly work. To address this gap, Glassick, Huber, & Maeroff (1997) proposed six standards (i.e., clear goals, adequate preparation, appropriate methods, significant results, effective presentation, and reflective critique) that could be applied equally to each kind of scholarship [12]. In 1999, Hutchings & Shulman coined the term *scholarship of teaching and learning* and articulated key differences between SoTL and scholarly teaching. Scholarly teaching was conceptualized as evidence-informed teaching; it draws on and implements insights from education-related literature to maximize the teacher’s effectiveness in the local context. In contrast, Hutchings & Shulman argued that SoTL requires the intentional study of educational efforts and the public dissemination of insights from such study so that others can build on and critique them [13]. Essentially, scholarly teaching focused on improving an *individual educator’s* teaching skills, while SoTL focused on helping the *field* advance.

While this framing has helped solidify the importance of SoTL in some HPE contexts, it seems to have been ineffective in others. Why would this dichotomy exist? Perhaps some contexts value SoTL activities because they rely on a specific definition of SoTL? Perhaps the outcomes of SoTL-activities are more readily recognized in some contexts? Perhaps the value of SoTL in P&T decisions has changed over time? We undertook this study to better understand assumptions, norms, and expectations about SoTL informing the HPE literature. To that end, we conducted a scoping review of the HPE literature to map how SoTL has been conceptualized, realized, and institutionally recognized by P&T committees. Knowing that SoTL-related institutional practices are fundamentally shaped by a nation’s academic settings and by national-level accreditation mandates, we concentrated our review on one national context: the United States of America.

## Methods

We conducted a scoping review because it supports the mapping of characteristics across a diverse body of publications [15,16]. We followed Arksey & O’Malley’s scoping review methodology [17], and incorporated stakeholder feedback as suggested by Levac et al. [18] The study adhered to the PRISMA-ScR checklist [19] (see Supplementary Material 1). In keeping with the constructivist roots of scoping reviews [20], we acknowledge that this synthesis was informed by our own professional experiences (see Supplementary Material 2 for reflexivity statements).

### Identifying the Research Question

The overarching question guiding this review was: *How has SoTL has been conceptualized, realized, and institutionally recognized in the HPE literature?* After preliminary review of the literature, we decided to add two sub-questions: *How has SoTL been described in relation to promotion and tenure processes in the HPE literature? How have these descriptions changed over time?*

### Identifying Relevant Articles

A research librarian (JL-M) executed a search in July 2024 and re-ran it in July 2025 and April 2026 (capturing additional, recent publications). The search was conducted across PubMed, Embase, CINAHL, PsychInfo, ERIC, Web of Science, and Scopus. The search strings included a combination of Medical Subject Headings (MeSH) terms, keywords, and search filters (see Supplementary Material 3 for the exact search carried out in each database). We defined SoTL broadly and so searched for articles referring to any scholarship relating to education, teaching, and/or learning (e.g., scholarship of teaching; educational scholarship; scholarship of teaching and learning). We were inclusive of articles from all HPE domains. No date range restrictions were imposed, but included articles needed to have been published in English. Results were exported to Covidence [21] and were deduplicated therein.

### Selecting the Publications to be Included

Three members of the research team (LV, KS, RE) met regularly to develop and refine inclusion and exclusion criteria to ensure alignment between the study’s research questions and the articles retrieved. Articles were retained if they were published in peer-reviewed journals, addressed SoTL in relation to the education of any healthcare professional, and had been conducted in the United States. We excluded articles if they: were published in a book, conference proceedings, or any other non-peer reviewed source; addressed a profession where care was not provided to humans (e.g., veterinary medicine) or was not related to health professions (e.g., general education); the first or senior author was not based at an American institution; or reported on local innovations (e.g., educational intervention reports labeled as a SoTL activity).

Article screening was conducted in two phases. First, three reviewers (LV, FJ, and JL-M) independently reviewed titles and abstracts against inclusion and exclusion criteria. If a collaborator was unsure if the article should be included, it was forwarded to full text review. Next, two collaborators (LV or FJ) reviewed the full text of each included article. At this stage two additional exclusion criteria were added to the study: articles were excluded if only passing reference to SoTL was made (e.g., a single, aside comment such as: “Study findings could inform SoTL considerations.”) or if SoTL (or a related term—e.g., educational scholarship) was not included. Any uncertainty regarding inclusion was resolved through discussion until consensus was reached. We hand searched the references of all articles that met our inclusion criteria to identify and consider for inclusion additional articles. See Supplementary Material 4 for screening workflow.

### Charting the Data

A data extraction tool was developed and piloted by LV, KS, and RE on 8 different articles, revising it to resolve discrepancies and increase clarity. It was then tested by LV, KS, and RE on 5 different articles from the corpus and adjusted accordingly. The final extraction tool is provided in Supplementary Material 5. Using this tool, seven research team members (FJ, LV, KS, JA, KD, GJ, RE) extracted data from each article. Two reviewers performed data extraction on each article to ensure interpretive alignment. Uncertainties and discrepancies were resolved by a third reviewer.

### Collating, Summarizing, and Reporting Results

We used descriptive statistics to summarize the definitions and conceptualizations of SoTL articulated in the articles, and the way SoTL had been operationalized (i.e., outcomes described as evidence of a clinician educator’s SoTL activity). We then used qualitative content analysis [22] for descriptive purposes (i.e., using an inductive approach with low abstraction) [23], to describe *if* (and when relevant *how*) SoTL activities had been described in relation to P&T and how those descriptions had changed over time.

### Consulting Stakeholders

Once preliminary findings were drafted, we solicited feedback from 9 stakeholders who had actively contributed SoTL expertise to the National Academies of Science, Engineering and Medicine’s Global Forum on Innovation in Health Professions Education and/or who had contributed to the SoTL-focused literature in HPE. Institutional ethical approval was sought; the study was deemed exempt. The stakeholders represented medicine (n = 3), physical therapy (n = 2), nursing (n = 1), dentistry (n = 1), occupational therapy (n = 1), and pharmacy (n = 1). Each stakeholder was presented with our preliminary findings, asked for reflections on the extent to which the findings aligned with their experiences, and asked for their reactions. Stakeholders confirmed the study findings, highlighting the impact of institutional culture on SoTL recognition. Building on this insight, we used the theory of Institutional Logics [24] to enhance our data interpretations.

## Results

**Figure 1:**
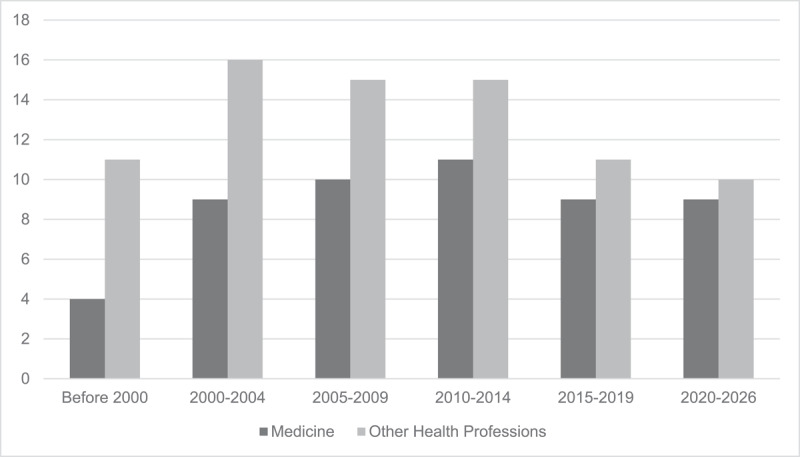
Publications grouped into 5-year blocks for articles in medicine and other health professions.

After deduplication, we identified 4,588 articles for consideration. We excluded 4,301 articles during title and abstract review, and an additional 157 at full text review, leaving 130 articles in the study corpus. No new articles were found via the hand search of article references. The HPE domains represented were medicine (n = 52; 40%) [3,5,7,8,9,25,26,27,28,29,30,31,32,33,34,35,36,37,38,39,40,41,42,43,44,45,46,47,48,49,50,51,52,53,54,55,56,57,58,59,60,61,62,63,64,65,66,67,68,69,70,71] and nursing (n = 42, 32%) [72,73,74,75,76,77,78,79,80,81,82,83,84,85,86,87,88,89,90,91,92,93,94,95,96,97,98,99,100,101,102,103,104,105,106,107,108,109,110,111,112,113], with all other domains making up the remaining 28% (n = 36) [114,115,116,117,118,119,120,121,122,123,124,125,126,127,128,129,130,131,132,133,134,135,136,137,138,139,140,141,142,143,144,145,146,147,148,149], (i.e., pharmacy [n = 15], dentistry [n = 3], physical therapy [n = 8], and occupational therapy [n = 5]). There were 5 articles [118,120,123,145,146] that covered both medicine and non-medical domains; these were grouped with the other domains in our analyses (see Supplementary Material 6 for the full list of corpus articles, grouped by profession and chronologically). Given that this manuscript addresses a medical education audience, we present the findings of medicine’s SoTL literature (n = 52) against the sum of findings for all other HPE domains (n = 78). We grouped articles into 5-year blocks to even out year-to-year variances and plotted the number of publications in each group (see Figure 1). SoTL-related publication frequency peaked in 2010–2014, with a steady slow decline thereafter.

### How SoTL was Conceptualized

The conceptualization of SoTL was consistent between articles from medicine and those from other disciplines. Most articles explicitly grounded their SoTL discussions in three seminal texts. Boyer (1990) [11] was cited in 88 articles (68%) (40 from medicine), Glassick et al. (1997)[12] were cited in 27 articles (21%) (18 from medicine), and Hutchings and Shulman (1999) [13] were cited in 24 articles (18%) (10 from medicine). Even when these theorists were not directly referenced, SoTL was described in ways that mirrored the Boyer, Glassick et al., and/or Hutchings & Shulman theories (i.e., descriptions adhered to those of the theorists but did not explicitly cite them). In sum, 82% of all articles (n = 106) either explicitly or implicitly conceptualized SoTL in alignment with one or more of these three seminal works (see Supplementary Material 7 for the full list of explicit and implicit references listed for each article).

Across these direct and indirect references, the articles consistently relied on the same aspects of each seminal SoTL resource. In relation to Boyer, the articles relied on this theorist’s discussion of teaching as a form of scholarship distinct from the scholarships of application, discovery, or integration [3,5,7,8,9,25,26,28,29,31,32,33,35,36,38,39,40,41,43,44,48,50,51,52,53,54,55,57,58,59,60,61,62,63,64,65,66,67,69,71,72,73,74,76,77,78,79,80,83,84,85,86,87,89,90,92,95,96,97,98,99,100,101,102,103,106,108,109,110,111,112,113,114,115,118,124,125,126,128,130,131,132,135,139,141,142,144,145,146,148,149]. With respect to Glassick et al.’s work, the articles celebrated the six standards for evaluating scholarship and framed them as appropriate markers of rigor for SoTL activities [7,9,25,26,28,29,31,32,33,34,35,38,41,50,51,52,53,62,66,69,80,94,95,106,107,114,115,120,121,130,132,133,134,135,136,137,138,141,147,149]. Finally, articles drawing on Hutchings and Shulman consistently highlighted their distinction between *scholarly teaching* and SoTL [25,32,33,36,39,47,48,51,53,56,62,67,70,92,99,100,104,107,110,120,125,126,130,131,135,137,141,142,144,145,147,148,149]. None of the articles in the corpus critiqued or suggested revisions to the conceptualizations laid out by Boyer, Glassick et al. and/or Hutchings & Shulman. The common use and unrevised adoption of these seminal works reflected a shared understanding of and position on SoTL across the HPE literature.

### How SoTL was Operationalized

We identified 35 distinct ways in which SoTL had been operationalized, which we organized into 12 categories (see Supplementary Material 8). Individual articles commonly listed more than one form, resulting in a total of 240 references to SoTL operationalizations in the corpus. Because a key distinction between SoTL and scholarly teaching is the intended audience of dissemination (i.e. SoTL targets broad audiences so that other scholars can build on the work, while scholarly teaching aims at a local audience), we organized the 12 categories into 3 groups that reflected the intended audience of the dissemination: public (e.g. peer-reviewed publications, presentations, books, grant funding), local (e.g. teaching, clinical practice, teaching portfolios, evidence-based teaching or evaluation strategies), or undetermined (e.g. new courses or curricula, peer recognition, developing educational resources, assessments). The undetermined categories could not be consistently labeled as directed towards public or private audiences because of the variability in how each operationalization was described in individual manuscripts (e.g., sometimes *awards/peer-recognition* described a student-nominated local award for teaching excellence [38], sometimes it was a national or international recognition for contributions to the HPE literature [122]; sometimes it was simply stated as “awards”) [32].

In total, 40 articles (31%) described SoTL operationalizations involving public dissemination (12 from medicine), while 45 articles (35%) described SoTL operationalizations directed towards local audiences (15 from medicine), and 49 articles (38%) described undetermined dissemination (17 from medicine). Table 1 presents a summary of SoTL operationalizations by group and category, and by health profession. Supplementary material 8 details operationalizations by group and category for each corpus manuscript.

**Table 1:** SoTL operationalizations by audience: public disseminations, local disseminations, and public or local disseminations.


	PUBLIC DISSEMINATION				LOCAL DISSEMINATION				UNDETERMINED (i.e., PUBLIC OR LOCAL) DISSEMINATION			
		
	PEER-REVIEWED PUBLICATIONS	PUBLIC PRESENTATIONS	BOOKS	GRANT FUNDING	TEACHING	CLINICAL PRACTICE	TEACHING PORTFOLIOS	EVIDENCE BASED TEACHING OR EVALUATION STRATEGIES	NEW COURSES/CURRICULA	AWARDS/PEER RECOGNITION	CREATING EDUCATIONAL RESOURCES	ASSESSMENT/EVALUATION

**MEDICINE**												

	12	3	2	1	9	2	8	1	11	3	10	11

**Medicine Totals**	**18**				**20**				**35**			

**OTHER HEALTH PROFESSIONS**												

**Nursing**	10	4	4	7	12	1	2	3	12	4	10	13

Nursing Totals	25				18				39			

**Pharmacy**	9	6	3	3	7	0	1	0	5	1	6	6

Pharmacy Totals	21				8				18			

**Physical Therapy**	0	0	0	0	1	0	1	1	1	0	0	1

Physical Therapy Totals	0				3				2			

**Occupational Therapy**	1	0	0	0	1	1	0	1	0	0	1	1

Occupational Therapy Totals	1				3				2			

**Dentistry**	2	2	0	0	1	0	0	0	0	0	1	0

Dentistry Totals	4				1				1			

**HPE/Mix**	3	2	1	2	2	1	1	0	2	2	3	2

HPE/Mix Totals	8				4				9			

**All Other Professions Total:**	**59**				**37**				**71**			

**Totals in Full Corpus**	**37**	**17**	**10**	**13**	**33**	**5**	**13**	**6**	**31**	**10**	**31**	**34**

	**77**				**57**				**106**			


The SoTL operationalizations that were directed towards local audiences would, according to Hutchings & Shulman’s definition, be better classified as examples of scholarly teaching. A portion of the undetermined-audience references to SoTL operationalizations could similarly be categorized as either examples of SoTL or of scholarly teaching.

### How SoTL Informed Promotion and Tenure

SoTL in the context P&T processes was mentioned in 58 articles (45%) (33 from medicine), with 15 articles (26%) describing how schools and academic teaching hospitals had revised P&T guidelines to recognize and/or support SoTL activities. However, many articles (n = 40; 69%) warned that recognizing and valuing SoTL contributions had been limited by P&T committees (with some articles noting limitations in more than one category). Specifically, 22 articles noted P&T committees were unfamiliar with SoTL, 25 described P&T processes that lacked mechanisms to judge SoTL activities and products, while 25 articles lamented that P&T committees upheld traditional research metrics for academic success (e.g., peer-reviewed publications, grant funding) that reflected the primacy of basic and/or clinical research. Several articles (n = 14) noted that quantifying the success of SoTL activities and products was difficult and suggested that this impeded the recognition of educators in P&T decisions. Table 2 lists all manuscripts relating to each of these P&T topics. Some articles (n = 15) addressed neither institutional supports nor obstructions.

**Table 2:** Descriptions of SoTL in relation to promotion and tenure across time in medical education and other health professions education literature.


		BEFORE 2000	2000–2004	2005–2009	2010–2014	2015–2019	2020–2026

Schools and academic teaching hospitals have revised their promotion and tenure guidelines to recognize SoTL	Medicine		Beattie 2000; Haffler 2000	Fincher 2006; Wood 2006; Schrader 2008; Ruiz 2009	Grigsby 2011; Searle 2012; Crites 2014	Kyle 2017; O’Brien 2019	

	Other HPE			Smith 2005; Spake 2005	Gupta 2014	Register 2018	

Promotion and tenure committees (or similar gatekeeping bodies) that were unfamiliar with SoTL	Medicine			Morahan 2007; McGaghie 2009; Ruiz 2009	Grigsby 2011; Crites 2014	Ander 2017; Kyle 2017; Franzen 2018; Irby 2018	Milner 2023

	Other HPE	Miller 1991	Reece 2001; Glanville 2004	Eddy 2007; Spath 2007; Peterson 2005; Smesny 2007; Spake 2005	Oermann 2014; Lanning 2014	Opacic 2017; Register 2018	

Promotion and tenure committees lacked mechanisms to judge the SoTL activities and products presented in promotion	Medicine		Haffler 2000; Simpson 2000; Collins 2004	Simpson 2007; Ruiz 2009	LaMantia 2010; Grigsby 2011; Shah 2012; Crites 2014	Ander 2017; Irby 2018	Hoffman 2020; Jacobs 2020; Blanco 2022; Bockrath 2024; Gribble 2026

	Other HPE		Glanville 2004	Eddy 2007; Smesny 2007; Wise 2008	McNeal 2014; Lanning 2014	Oermann 2017; Opacic 2017; Register 2018	

Promotion and tenure committees upheld traditional metrics for academic success (e.g., peer-reviewed publications, grant funding) that reflected the primacy of research	Medicine		Beattie 2000; Haffler 2000; Smith 2001	Schrader 2008; McGaghie 2009	Greenberg 2010; Grigsby 2011; Searle 2012; Crites 2014	Jordan 2016; Kyle 2017; Franzen 2018; Irby 2018	Jacobs 2020; Beck Dallaghan 2023

	Other HPE	Starck 1996; Everett 1998; Sherwen 1998	Riley 2002	Stull 2005; Wise 2008	McNeal 2014; Oermann 2014; Jahangiri 2011	Mehvar 2017	

Quantifying the success of SoTL activities and products was very difficult, and they suggested that this impeded the recognition of physician educators via promotion and tenure decisions	Medicine	Simpson 1999; Schneeweiss 1997	Beattie 2000; Simpson 2000; Collins 2004	McGaghie 2009		Irby 2018	Jacobs 2020

	Other HPE	Angstadt 1998	Glanville 2004	Spath 2007	McNeal 2014; Oermann 2014	Register 2018	


We mapped descriptions of SoTL in relation to P&T across a timeline (see Table 2) and noted some apparent trends. In the medical education literature, descriptions of how SoTL gained institutional acceptance and recognition started in 2000–2004, peaked between 2005–2014, declined between 2015–2019, and were absent since 2020. In contrast, descriptions of obstructions impeding institutional acceptance and recognition of SoTL (i.e., P&T committees being unfamiliar with SoTL, lacking mechanisms for judging SoTL, or upholding traditional research metrics for success, and having difficulty quantifying success of SoTL activities) began before 2000 but grew quickly and has remained an issue throughout the period of time covered in this review.

In the literature relating to other health professions, descriptions of how SoTL was gaining institutional acceptance and recognition began in 2005 but was minimally present from 2011–2019. In contrast, descriptions of obstructions impeding institutional recognition of SoTL began before 2000 and grew over time, being consistently present in the literature until 2015–2019. We found no descriptions of SoTL in relation to P&T—neither enabling nor obstructing—for other health professions since 2019.

## Discussion

The Boyer [11], Glassick et al. [12], and Hutchings & Shulman [13] conceptualizations of SoTL have been consistently adopted in the HPE literature without revision or adaptation. Categorizing the SoTL operationalization into 3 groups differentiated by intended audience revealed that a broad range of operationalizations were presented in the corpus. The literature reports that SoTL is inconsistently acknowledged and valued across HPE institution’s P&T committees, with 28/58 articles (48%) noting how P&T committees struggled to acknowledge, assess, and/or quantify SoTL activities. These findings do not explain why SoTL is variably recognized and valued across America’s HPE institutions; however, they do offer insights into the values and norms that may underpin this inconsistency. To ground this analysis in theory, we harness the theory of Institutional Logics.

### Institutional Logics

Institutional logics proposes that individual organizations (e.g., medical schools, academic teaching hospitals) within a broad social group (e.g., HPE in the United States of America) uphold a shared rationality because they maintain similar socially validated understandings and assumptions (i.e., institutional logics) [150]. Institutional logics are the socially constructed and historically informed patterns of practices, assumptions, values, and rules through which individuals produce (and reproduce) meaning for their social reality and through which they justify their actions [151]. When applied to HPE in America, we can interpret Boyer’s argument for the recognition of four kinds of scholarship as a push against a shared institutional logic that positioned research as academia’s dominant logic. Most organizations, including America’s HPE organizations, house multiple institutional logics—e.g., Boyer asserted that four logics (represented by the scholarships of research [i.e., discovery], teaching, integration, and application) are present in academia. In some organizations, multiple logics can generate contestation and conflict [152,153]; in others, multiple logics can coexist harmoniously [154]. This explains the variability of SoTL recognition across American HPE organizations—i.e., while organizations can house similar logics, the ways those logics are internally negotiated generates unique outcomes for each institution [155].

**Figure 2:**
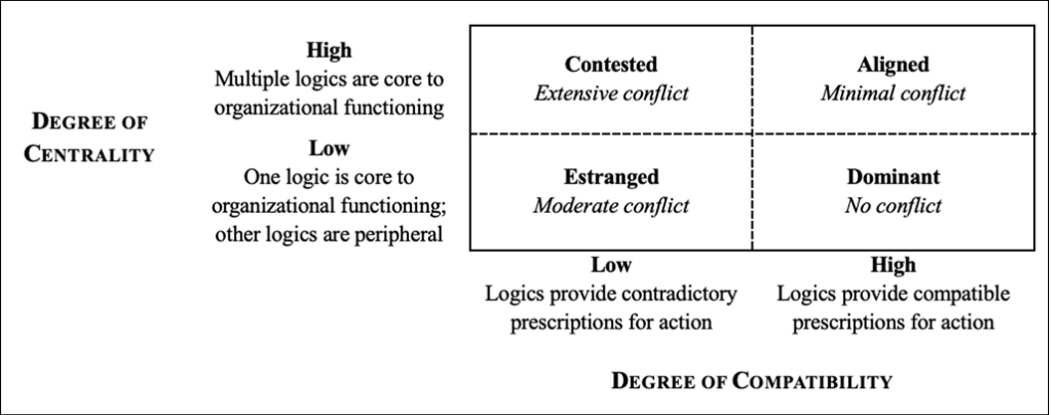
Besharov and Smith’s (2014) Type of Logic Multiplicity Within Organizations.

Besharov and Smith [156] created a model explaining why multiple logics generate conflict in some organizations but are compatible in others. They tied this variability to how logics are realized within an organization, proposing that two dimensions shape each incorporation: *compatibility* and *centrality* [156]. Compatibility is defined as the extent to which institutional logics are consistent and mutually reinforcing [156]. High compatibility means that logics are coherent, providing congruent rules for action; low compatibility means that logics are inconsistent, providing incongruent rules for action. Centrality is defined as the degree to which multiple institutional logics are treated as equally valid and relevant to the organization’s functioning [156]. High centrality means that multiple logics are recognized as core to the organization’s functioning; low centrality means that one logic is considered core and others are peripheral to the organization’s functioning. Compatibility and centrality can be visualized as existing in a 2 × 2 table (see Figure 2), wherein each cell is represented with an ideal type of organization (i.e., *contested* [i.e., extensive conflict]; *aligned* [i.e., minimal conflict]; *estranged* [i.e., moderate conflict]; *dominant* [i.e., no conflict]). The dashed lines between cells illustrates that compatibility and centrality are continuous dimensions; therefore, organizations can exist between ideal types.

### Applying Institutional Logics Theory to Review Findings

To understand why SoTL’s importance seems to have been confirmed in some HPE contexts but not in others, we can consider how the logics of research and SoTL have been variably incorporated into the organizations represented in the literature. Since the logic of research has traditionally been celebrated in academia, it is noteworthy that several articles described contexts where P&T committees revised guidelines to recognize SoTL activities. This suggests that these organizations have allocated high centrality to both the logics of research and SoTL (i.e., both are important to the institutions’ functioning), and that they created high compatibility (i.e., congruent rules for P&T decisions for both logics). Given the high centrality and high compatibility of the logics, these contexts likely act as *aligned* organizations and so have minimal conflict between the logics of research and SoTL.

In contrast, many articles identified factors impeding the recognition of SoTL in P&T decisions, including P&T committees’ practice of applying research’s metrics for success on SoTL activities. We propose two possible interpretations of these data. First, these data may suggest that, in contexts where such impeding factors exist, logics have low centrality (i.e., that the logic of research is considered central to organizational functioning, while the logic of SoTL is peripheral) and low compatibility (i.e., the logics of research and SoTL provide contradictory rules for action [e.g., the rules for recognizing research are not aligned with those for recognizing SoTL]). These contexts would be examples of *estranged* settings where moderate conflict between the logics exists. Alternatively, it may be that, in these settings, there is high centrality (i.e., that both logics are institutionally valued) but low combability (i.e., research and SoTL have incongruent rules for action). If this is the case, these contexts would exemplify *contested* organizations wherein extensive conflict between the logics of research and SoTL exist.

We propose that the degree to which the logic of SoTL is integrated—in terms of centrality and compatibility—with the logic of research impacts the extent to which SoTL is institutionally recognized and valued. This would explain why some American HPE institutions are supportive of SoTL activities, while others are far less so. It should be noted that some articles listed both supportive and obstructing factors (e.g., Kyle, 2017) [47], underscoring the point that centrality and compatibility exist in degrees and so organizations can fall between ideal types.

SoTL’s variable recognition in different HPE institutions is also likely influenced by the extent to which a logic’s prescriptions for action (i.e., compatibility) are clearly articulated. Determining the degree of compatibility between institutional logics requires comparing the prescriptions for action for each logic. The beliefs and practices of the logic of research are broadly known and accepted (e.g., a faculty member’s research success can be measured, in part, by the number of high-impact peer-reviewed publications disseminated). However, despite the common adoption of the Boyer [11], Glassick et al. [12], and Hutchings & Shulman [13] traditions, there remains considerable confusion in the American HPE literature about what SoTL *actually is*. This is evident in our findings related to SoTL operationalizations: the public operationalizations of SoTL mirror those of the logic of research (i.e., peer-reviewed publications, public presentations, books, and grant funding) and the local operationalizations align with scholarly teaching (i.e., teaching, clinical practice, teaching portfolios, evidence-based teaching or evaluation strategies). Which operationalizations are uniquely attributable to SoTL remains unclear, making it difficult for organizations to create compatibility and for scholars to account for SoTL activity. This likely also contributes to P&T committees’ reliance on research metrics to assess SoTL: in the absence of clear SoTL expectations, these committees rely on the logic they know best—i.e., the logic of research.

### Implications

Institutional Logics can provide insights into how the recognition of SoTL in an organization might be changed. Institutional logics can be shaped and changed at the individual, organizational, and field levels [24]. First, changes in the value of an institutional logic can be realized by the actions of individuals. Many HPE scholars have worked to elevate the logic of SoTL in American HPE contexts; institutional logics would label these individuals as *institutional entrepreneurs* (i.e., actors who have an interest in and take responsibility for transforming an organization, either in full or in part) [157]. In contexts where the logic of SoTL is recognized, one or several institutional entrepreneurs likely intentionally increased the value and codified recognition of the logic of SoTL thereby transforming the balance of logics in their institution. At institutions where SoTL has yet to find organizational recognition, an institutional entrepreneur is likely needed to champion and realize similar changes. Research suggests that there aren’t specific criteria establishing who will be a successful institutional entrepreneur; instead, success is tied to an individual’s ability “to see or create ‘a window of opportunity’” [157 (p. 202)] and to leverage resources towards seizing that opportunity. Thus, for the logic of SoTL to be valued in an American HPE institutions (including P&T committees), someone is needed who can see (and/or who is willing to create) an opportunity to effect this change *and* who is socially positioned with authority, skills, and knowledge to bring about this change.

Second, organization-level practices can impact the centrality of an institutional logic. An organization’s hiring practices and internal socialization efforts shape who is part of the organization and the nature of the logics those individuals value [156]. If an HPE organization consistently hires individuals who support the logic of SoTL and socializes individuals within the organization to uphold the logic of SoTL, then SoTL can gain institutional value, becoming more central to the organization over time. This strategy requires a long-term commitment (i.e., it can take years for individuals and socialization efforts to permeate a context), but it can be intentionally realized. Other strategies rely on changes in broader circumstances (e.g., shifts in resource availability [e.g., a new high dollar-value SoTL-focused HPE grant] or modifications in regulations [e.g., an accreditation requirement for HPE organizations to support SoTL-focused careers]). While such broader changes can create a new balance of institutional logics [158,159], realizing these shifts is usually beyond the purview of individual organizations.

Finally, the field can bolster the uptake of the logic of SoTL by clearly articulating SoTL’s prescriptions for actions (i.e., compatibility structures). Boyer framed *scholarship* as a superordinate, umbrella term encompassing “a variety of creative work carried on in a variety of places,” [11 (p. 15)] and argued for the recognition of four types of scholarship (i.e., discovery, integration, application, and teaching). Boyer offered a comprehensive and dynamic understanding of scholarship, wherein “the rigid categories of teaching, research, and service are broadened and more flexibly defined.” [11 (p. 16)] While this expansive definition has historically supported SoTL’s recognition in HPE, its breadth has become problematic. As our analysis illustrates, an incredible scope of operationalizations (including scholarly teaching activities and educational research outputs) has been identified as evidence of SoTL. We propose that this dilutes the meaning of scholarship and, more specifically, SoTL. How can the logic of SoTL—especially its compatibility structures—be championed at P&T committees when scholarship can encompass such variety? We propose that distinguishing between scholarly teaching, SoTL, and educational research can help to reveal the logic of SoTL and thereby support compatibility.

To realize this goal, we started with literature from our corpus, specifically Medina et al’s (2011) framework [137]. We augmented it by interpreting and applying: (a) Milner et al’s (2023) three essential components for scholarship—advancement of knowledge, dissemination, impact [52]; (b) operationalization examples from our review; and (c) definitions and descriptions for each term from a range of sources (i.e., both within and external to the study corpus). We filled gaps in the framework by drawing on the insights we developed through this literature review. This amalgamation resulted in a new framework distinguishing between scholarly teaching, SoTL, and educational research (see Table 3).

**Table 3:** The differences between scholarly teaching, the scholarship of teaching and learning, and educational research.


	SCHOLARLY TEACHING	SCHOLARSHIP OF TEACHING AND LEARNING	EDUCATIONAL RESEARCH

Definition	“Scholarly teaching is teaching that is well grounded in the sources and resources appropriate to the field. It reflects a thoughtful selection and integration of ideas and examples, and well-designed strategies of course design, development, transmission, interaction and assessment” [160 (p. 49)]. “Scholarly teaching relates to using the work produced by others (the literature) to inform and guide one’s practice and the teaching-learning experience” [161 (p. 825)].	SoTL is “a broad set of practices that engage teachers in looking closely and critically at student learning in order to improve their own courses and programs, and to share insights with other educators who can evaluate and build on their efforts” [1 (p. xix)]. SoTL “advances the profession of teaching itself”, by using SoTL activities “at the level of institutional and national policy, to develop competence, excellence, and promotion frameworks” [161 (p. 825)]. SoTL can address topics that may not be easily categorized as *teaching* or *learning* (e.g., medical school admissions; specialty selection; clinician educator career paths)	Educational research creates and disseminates “better ways of thinking about the problems we face [in HPE] … the value of our scientific discourse (our talks and papers) will arise not from our ability to create a general solution that will apply to everyone’s problems or even our ability to solve each other’s problems, but rather from our ability to help each other think better about our own versions of the problems” [162 (p. 37)].

Key features	Scholarly teaching “promotes student engagement and learning using the educational literature and systematically assesses learning outcomes” [137 (p. 257)]. “Scholarly teaching *draws* on the expertise of others” [161 (p. 825)].	SoTL “communicates the goals, preparation, methods, results, presentation, and reflection of teaching in the literature” [137 (p. 257)]. “SoTL *contributes to* the expertise of others” [161 (p. 825)].	Educational research uses research designs—be they quantitative, qualitative, or mixed—to study educational questions of broad relevance [137]. Educational research contributes new knowledge to HPE’s body of literature.

Audience for Disseminations	Local audience (i.e., improving teaching and learning within the local context). “Sharing is aimed at enhancing the value of teaching at the institution as well as developing and supporting individuals in their subsequent teaching practice” [161 (p. 825)].	Public audience (i.e., HPE educators external to the local context; may be of interest to HPE researchers)	Public audience (i.e., broad audience of all external HPE educators and HPE researchers)

Evidence [137] and disseminations [52]	Teaching portfolio [137]; teaching; clinical practice [including workplace-based teaching]; teaching evaluations; implementing evidence-based teaching, course creation, or evaluation strategies; local awards recognizing teaching excellence	Peer-reviewed publications presenting instructional design or assessment insights; local, national, or international talks on educations [137]; program evaluation with broadly transferable findings; reporting of educational resources with evaluation data	Peer-reviewed publications presented as research studies in educational literature [137]; peer-reviewed conference presentations and abstracts; authoring books and/or book chapters; securing of grant funding; awards for contributions to HPE’s knowledge base.

Advancement of knowledge [52]	Harnessing knowledge about how teaching educations learners/transmits knowledge to learners. The learners’ knowledge is advanced. The educator’s skill are improved. Advancements are locally focused.	Development and assessment of educational approaches, curricula, innovations, etc. for the transmission of knowledge to learners. The knowledge in the HPE field about how to effectively engage in HPE is advanced.	Discovery of new knowledge through investigation [52] (i.e., research); integration of knowledge to generate new understanding [52] (i.e., literature reviews/syntheses); knowledge and understanding about topics impacting HPE are advanced [161].

Impact [52]	Measurable impact at the local level (e.g., student learning is enhanced by the educator’s teaching and/or evaluation practices) [52].	Measurable impact at the local, national, and/or international level (e.g., an educational innovation developed by an educator is adopted across their local institution, and/or other institutions nationally, and/or institutions in other countries)	Measurable impact at the national and/or international level (e.g., a research discovery influences the direction of the HPE field or provides a platform for others to build on; a meta-analysis integrates a body of knowledge for new insights, policies, or guidelines) [52].

Illustrative example	The educator learns about/reads into/takes training to use Team Based Learning. They implement it in their classroom activities to integrate foundational and clinical science knowledge and skills to diagnose and manage a patient with dyslipidemia. Student evaluations reveal greater engagement with the material and greater enjoyment of classroom time. The educator reports this work in their teaching portfolio.	The educator analyzes their TBL teaching activity (focused on integrating foundational and clinical science knowledge and skills to diagnose and manage a patient with dyslipidemia), and communicates the insights gained via this analysis by reporting the goals, preparation, methods, results (e.g., student performance outcomes; student feedback to the educator), and reflections on/interpretations of their teaching experiences. The educator publishes this work [163] in MedEd Portal, a publicly available repository of stand-alone, complete teaching or learning modules.	The researcher investigates an offering of TBL in their local institution to explore how many changes can be made to an educational intervention (i.e., TBL) to still be considered an example of that intervention. This research proposes distinguishing between the techniques, principles, and philosophy of the intervention, and theorizes that techniques can vary wildly but, if the intervention stays true to the principles and philosophy of the intervention, it remains an example of that intervention. This model (techniques vs principles vs philosophy) is offered as a way of understanding the conceptual structure of any educational intervention [164]. It is published in a peer reviewed journal.


### Limitations

Like all literature reviews, our synthesis was limited by what has been published. While the number of publications addressing SoTL is decreasing, we do not interpret this decline as an indication of the waning importance of SoTL in HPE; instead, we suggest that it reflects how the legitimacy of SoTL has been solidified in some American HPE organizations but fallen off the radar of others. The waning body of literature may reflect a *que será será* (whatever will be, will be) fixed mindset. However, for SoTL-focused activities to be more universally valued, a new wave of effort is needed from individuals, organizations, and the HPE field to support a change in the valuing of institutional logics across American settings. Second, our review focused only on American literature; this leant the study coherence at the possible cost of more internationally generalizable findings. While this is a limitation, using the theory of Institutional Logics enhances transferability via theoretical engagement thereby supporting the relevance of our findings to other contexts at a conceptual level [165]. Next, we conducted a review of existing literature; we did not seek out definitions and conceptualizations of SoTL or of P&T policies related to SoTL from individual HPE institutions. Future research should examine these documents to see if they (mis)align with the published literature. Finally, our team’s unique composition shaped our study design, data extraction foci, and interpretations. Other researchers might have relied on other theories, highlighted other data, or explained them differently.

## Conclusion

This scoping review’s use of Institutional Logics to analyze the conceptualizations, operationalizations, and framing of SoTL in relation to P&T processes suggests that the logics of research and SoTL are aligned (vis a vis centrality and compatibility) in some contexts but misaligned in others. We propose that alignments can be achieved in individual contexts but realizing that goal will require individual-, organizational-, and field-level efforts. In support of these efforts, we articulate the differences between scholarly teaching, SoTL, and educational research. These distinctions are essential because, if we can’t clearly define and explain SoTL, then we risk diminishing the value of the very thing that has bolstered the institutional recognition of education-focused careers in HPE in America.

## Additional Files

10.5334/pme.2740.s1 Supplementary Material 1. Preferred Reporting Items for Systematic reviews and Meta-Analyses extension for Scoping Reviews (PRISMA-ScR) Checklist.

10.5334/pme.2740.s2 Supplementary Material 2. Reflexivity Statement.

10.5334/pme.2740.s3 Supplementary Material 3. Search Strategies.

10.5334/pme.2740.s4 Supplementary Material 4. Screening Workflow.

10.5334/pme.2740.s5 Supplementary Material 5. SOTL in ME: Data Extraction Tool.

10.5334/pme.2740.s6 Supplementary Material 6. Corpus Articles.

10.5334/pme.2740.s7 Supplementary Material 7. Explicit and Implicit Reference to Seminal SOTL Scholars by Article.

10.5334/pme.2740.s8 Supplementary Material 8. SOTL Operationalizations by Audience Dissemination Focus.
