# Supplementary material for: A Scoping Review of the Conceptualization, Operationalization, and Institutional Recognition of the Scholarship of Teaching and Learning in Health Professions Education: Using Institutional Logics to Understand Inconsistencies
Source: Perspect Med Educ. 2026 Jun 5;15(1):482–501. doi: 10.5334/pme.2740 (PMC13239391; doi:10.5334/pme.2740)
Supplement: Supplementary Material 2. — Reflexivity Statement. [file pme-15-1-2740-s2.pdf]

Supplementary Material 2

Reflexivity Statement

The members of the research team hold a varied set of positions in relation to SoTL. Five team members are trained health professionals from three different professions (medicine [KS, JA], physical therapy [KD, GMJ], and nursing [PRJ]). Two collaborators are PhD-trained researchers specializing in HPE [LV, RE]. Rounding out the team are an academic librarian [JL-M] and a qualitatively trained research assistant [FJ]. Members of the research team had different levels of familiarity with the concept of SoTL when this review was initiated (varying from expert [e.g., LV, JA, KD, GMJ, PRJ, RE] to introductory [e.g., FJ, JL-M, KS]). Team members also hold a variety of positions in HPE-focused institutions—e.g., a Vice-Provost; a Dean; Directors in clinical contexts; Professors in clinical contexts; Researchers in academic and/or clinical contexts. Thus, the research team represented a wide range of viewpoints and experiences in relation to SoTL (e.g., academic leadership, faculty members, administrative staff). This diversity created several points of divergence in the interpretation of the literature. We regularly reflected on how our training, institutional roles, local contexts, and personal experiences informed our data interpretations. We engaged in debates about how aspects of the data (mis)aligned with our individual, personal experiences.
