# Supplementary material for: A Scoping Review of the Conceptualization, Operationalization, and Institutional Recognition of the Scholarship of Teaching and Learning in Health Professions Education: Using Institutional Logics to Understand Inconsistencies
Source: Perspect Med Educ. 2026 Jun 5;15(1):482–501. doi: 10.5334/pme.2740 (PMC13239391; doi:10.5334/pme.2740)
Supplement: Supplementary Material 3. — Search Strategies. [file pme-15-1-2740-s3.pdf]

Supplementary Material 3

Search Strategies

PubMed/Medline (Advanced search via https://pubmed.ncbi.nlm.nih.gov) - run July 24, 2024

#1: ("education, medical"[MeSH Terms] OR "education, nursing"[MeSH Terms] OR "education, dental"[MeSH Terms] OR "schools, medical"[MeSH Terms] OR "schools, dental"[MeSH Terms] OR "schools, nursing"[MeSH Terms] OR "schools, pharmacy"[MeSH Terms] OR "faculty, dental"[MeSH Terms] OR "faculty, medical"[MeSH Terms] OR "faculty, nursing"[MeSH Terms] OR "faculty, pharmacy"[MeSH Terms] OR "students, medical"[MeSH Terms] OR "students, dental"[MeSH Terms] OR "students, nursing"[MeSH Terms] OR "students, pharmacy"[MeSH Terms] OR "health professions school*"[Title/Abstract] OR "dental school*"[Title/Abstract] OR "dental student*"[Title/Abstract] OR "dentistry school"[Title/Abstract] OR "medical college*"[All Fields] OR "medical program*"[Title/Abstract] OR "medical school*"[Title/Abstract] OR "medical student*"[Title/Abstract] OR "medical teaching"[Title/Abstract] OR "medical training"[Title/Abstract] OR "nursing school*"[Title/Abstract] OR "nursing student*"[Title/Abstract] OR "pharmaceutical student*"[Title/Abstract] OR "pharmacy college*"[Title/Abstract] OR "pharmacy school*"[Title/Abstract] OR "pharmacy student*"[Title/Abstract] OR "physiotherapy student*"[Title/Abstract] OR "allied health student*"[Title/Abstract])

#2: ("Faculty"[MeSH Terms] OR "Teaching"[MeSH Terms] OR "Universities"[MeSH Terms] OR "educat*"[Title/Abstract] OR "facult*"[Title/Abstract] OR "pedagog*"[Title/Abstract] OR "professor*"[Title/Abstract] OR "teaching*"[Title/Abstract] OR "universit*"[Title/Abstract]) AND ("Allied Health Occupations"[MeSH Terms] OR "Dental Hygienists"[MeSH Terms] OR "Dentists"[MeSH Terms] OR "Nurses"[MeSH Terms] OR "Nursing"[MeSH Terms] OR "Nutritionists"[MeSH Terms] OR "Pharmacists"[MeSH Terms] OR "Physical Therapists"[MeSH Terms] OR "Physicians"[MeSH Terms] OR "Social Workers"[MeSH Terms] OR "allied health"[Title/Abstract] OR "audiolog*"[Title/Abstract] OR "dental hygienist*"[Title/Abstract] OR "dental"[Title] OR "dentist*"[Title/Abstract] OR "dieti*"[Title/Abstract] OR "endodontist*"[Title/Abstract] OR "health profession*"[Title/Abstract] OR "medical"[Title] OR "nurse"[Title/Abstract] OR "Nurses"[Title/Abstract] OR "Nursing"[Title] OR "nutritionist*"[Title/Abstract] OR "orthodontist*"[Title/Abstract] OR "periodontist*"[Title/Abstract] OR "pharmac*"[Title/Abstract] OR "physical therap*"[Title/Abstract] OR "physician*"[Title/Abstract] OR "physiotherapist*"[Title/Abstract] OR "social worker*"[Title/Abstract])

#3: ("scholarship teaching"[Title/Abstract:~3] OR "scholarship learning"[Title/Abstract:~3] OR "scholarship educational"[Title/Abstract:~3] OR "scholarship"[Title])

#4: (#1 OR #2) AND (#3)

#5: #4 AND (english[Filter])

Embase (Advanced Search via Embase) - run July 24, 2024

#1: ('allied health education'/de OR 'allied health student'/de OR 'dental education'/de OR 'medical education'/de OR 'medical school'/de OR 'medical student'/exp OR 'nursing education'/exp OR 'nursing student'/exp OR 'pharmacy school'/de OR 'physician assistant education'/de OR 'dental student'/de OR 'dental hygiene student'/de OR 'pharmacy student'/de OR 'health professions school*':ti,ab OR 'allied health student*':ti,ab OR 'dental school*':ti,ab OR 'dental student*':ti,ab OR 'dentistry school':ti,ab OR 'medical college*':ti,ab OR 'medical program*':ti,ab OR 'medical school*':ti,ab OR 'medical student*':ti,ab OR 'medical teaching':ti,ab OR 'medical training':ti,ab OR 'nursing school*':ti,ab OR 'nursing student*':ti,ab OR 'pharmaceutical student*':ti,ab OR 'pharmacy college*':ti,ab OR 'pharmacy school*':ti,ab OR 'pharmacy student*':ti,ab OR 'physiotherapy student*':ti,ab)

#2: ('teaching'/exp OR 'university'/de OR 'educat*':ti,ab OR 'facult*':ti,ab OR 'pedagog*':ti,ab OR 'professor*':ti,ab OR 'teaching*':ti,ab OR 'universit*':ti,ab) AND ('dental hygienist'/de OR 'dentist'/exp OR 'dietitian'/de OR 'nurse'/exp OR 'nursing'/exp OR 'pharmacist'/de OR 'physician'/exp OR 'physiotherapist'/de OR 'social worker'/de OR 'allied health':ti,ab OR 'audiolog*':ti,ab OR 'dental hygienist*':ti,ab OR 'dental':ti OR 'dentist*':ti,ab OR 'dieti*':ti,ab OR 'endodontist*':ti,ab OR 'health profession*':ti,ab OR 'medical':ti OR 'nurse':ti,ab OR 'nurses':ti,ab OR 'nursing':ti OR 'nutritionist*':ti,ab OR 'orthodontist*':ti,ab OR 'periodontist*':ti,ab OR 'pharmac*':ti,ab OR 'physical therap*':ti,ab OR 'physician*':ti,ab OR 'physiotherapist*':ti,ab OR 'social worker*':ti,ab)

#3: ((('educational' OR 'learning' OR 'teaching') NEAR/3 'scholarship'):ti,ab) OR 'scholarship':ti

#4: (#1 OR #2) AND (#3)

#5: #4 AND [english]/lim

CINAHL (Advanced Search via EBSCO)- run July 24, 2024

S1: ((MH "Education, Medical+") OR (MH "Education, Nursing+") OR (MH "Education, Pharmacy") OR (MH "Education, Allied Health+") OR (MH "Education, Dental") OR (MH "Schools, Medical") OR (MH "Schools, Nursing") OR (MH "Schools, Dental") OR (MH "Schools, Allied Health") OR (MH "Faculty, Dental") OR (MH "Faculty, Medical") OR (MH "Faculty, Nursing") OR (MH "Faculty, Allied Health") OR (MH "Students, Medical") OR (MH "Students, Dental") OR (MH "Students, Nursing+") OR (MH "Students, Pharmacy") OR (MH "Education, Allied Health") OR (MH "Students, Allied Health+") OR (MH "Education, Physician Assistants") OR (MH "Students, Dental Hygiene") OR TI ("health professions school*") OR AB ("health professions school*") OR TI ("Dental School*") OR AB ("Dental School*") OR TI ("dental student*") OR AB ("dental student*") OR TI ("dentistry school") OR AB ("dentistry school") OR TI ("medical college*") OR AB ("medical college*") OR TI ("medical program*") OR AB ("medical program*") OR TI ("medical school*") OR AB ("medical school*") OR TI ("medical student*") OR AB ("medical student*") OR TI ("medical teaching") OR AB ("medical teaching") OR TI ("medical training") OR AB ("medical training") OR TI ("Nursing School*") OR AB ("Nursing School*") OR TI ("nursing student*") OR AB ("nursing student*") OR TI ("pharmaceutical student*") OR AB ("pharmaceutical student*") OR TI ("pharmacy college*") OR AB ("pharmacy college*") OR TI ("Pharmacy School*") OR AB ("Pharmacy School*") OR TI ("pharmacy student*") OR AB ("pharmacy student*") OR TI ("Physiotherapy student*") OR AB ("Physiotherapy student*") OR TI ("allied health student*") OR AB ("allied health student*"))

S2: (((MH "Faculty") OR (MH "Teaching") OR (MH "Colleges and Universities") OR TI ("educat*") OR AB ("educat*") OR TI ("Facult*") OR AB ("Facult*") OR TI ("Pedagog*") OR AB ("Pedagog*") OR TI ("Professor*") OR AB ("Professor*") OR TI ("teaching*") OR AB ("teaching*") OR TI ("universit*") OR AB ("universit*")) AND ((MH "Allied Health Personnel") OR (MH "Dental Hygienists") OR (MH "Dentists+") OR (MH "Dietitians") OR (MH "Nurses+") OR (MH "Nutritionists") OR (MH "Pharmacists") OR (MH "Physical Therapists+") OR (MH "Physicians+") OR (MH "Social Workers") OR TI ("allied health") OR AB ("allied health") OR TI ("audiolog*") OR AB ("audiolog*") OR TI ("dental hygienist*") OR AB ("dental hygienist*") OR TI ("dental") OR TI ("dentist*") OR AB ("dentist*") OR TI ("dieti*") OR AB ("dieti*") OR TI ("endodontist*") OR AB ("endodontist*") OR TI ("health profession*") OR AB ("health profession*") OR TI ("medical") OR TI ("nurse") OR AB ("nurse") OR TI ("Nurses") OR AB ("Nurses") OR TI ("Nursing") OR TI ("nutritionist*") OR AB ("nutritionist*") OR TI ("orthodontist*") OR AB ("orthodontist*") OR TI ("periodontist*") OR AB ("periodontist*") OR TI ("pharmac*") OR AB ("pharmac*") OR TI ("physical therap*") OR AB ("physical therap*") OR TI ("physician*") OR AB ("physician*") OR TI ("Physiotherapist*") OR AB ("Physiotherapist*") OR TI ("social worker*") OR AB ("social worker*")))

S3: (MH "Scholarship") OR TI (scholarship N3 educational) OR AB (scholarship N3 educational) OR TI (scholarship N3 learning) OR AB (scholarship N3 learning) OR TI (scholarship N3 teaching) OR AB (scholarship N3 teaching) OR TI ("scholarship")

S4: (S1 OR S2) AND (S3)

S5: S4 Limits applied (Narrow by Language: English)

PsycInfo (Advanced Search - APA PsycInfo® via ProQuest) - run July 24, 2024

[S1]: [STRICT](MAINSUBJECT.EXACT("Medical Education") OR MAINSUBJECT.EXACT("Nursing Education") OR MAINSUBJECT.EXACT("Dental Education") OR MAINSUBJECT.EXACT("Medical Students") OR MAINSUBJECT.EXACT("Dental Students") OR MAINSUBJECT.EXACT("Nursing Students") OR tiab("health professions school*") OR tiab(("dental school" OR "dental schools")) OR tiab(("dental student" OR "dental students")) OR tiab("dentistry school") OR tiab(("medical college" OR "medical colleges")) OR tiab(("medical program" OR "medical programme" OR "medical programmes" OR "medical programs")) OR tiab(("medical school" OR "medical schools")) OR tiab(("medical student" OR "medical students")) OR tiab("medical teaching") OR tiab("medical training") OR tiab(("nursing school" OR "nursing schools")) OR tiab(("nursing student" OR "nursing students")) OR tiab(("pharmaceutical students")) OR tiab(("pharmacy college" OR "pharmacy colleges")) OR tiab(("pharmacy school" OR "pharmacy schools")) OR tiab(("pharmacy student" OR "pharmacy students")) OR tiab("Physiotherapy student*") OR tiab("allied health student*"))

[S2]: [STRICT] ((MAINSUBJECT.EXACT("Educational Personnel") OR MAINSUBJECT.EXACT("Teaching") OR MAINSUBJECT.EXACT("Colleges") OR tiab("educat*") OR tiab("Facult*") OR tiab("Pedagog*") OR tiab("Professor*") OR tiab("teaching*") OR tiab("universit*")) AND (MAINSUBJECT.EXACT.EXPLODE("Allied Health Personnel") OR MAINSUBJECT.EXACT("Dentists") OR MAINSUBJECT.EXACT.EXPLODE("Nurses") OR MAINSUBJECT.EXACT("Nursing") OR MAINSUBJECT.EXACT("Nutritionists") OR MAINSUBJECT.EXACT("Pharmacists") OR MAINSUBJECT.EXACT("Physical Therapists") OR MAINSUBJECT.EXACT.EXPLODE("Physicians") OR MAINSUBJECT.EXACT.EXPLODE("Social Workers") OR MAINSUBJECT.EXACT("Physician Assistants") OR MAINSUBJECT.EXACT("Medical Personnel") OR MAINSUBJECT.EXACT("Health Personnel") OR tiab("allied health") OR tiab("audiolog*") OR tiab("dental hygienist*")

OR ti("dental") OR tiab("dentist*") OR tiab("dieti*") OR tiab("endodontist*") OR tiab("health profession*") OR ti("medical") OR tiab("nurse") OR tiab("Nurses") OR ti("Nursing") OR tiab("nutritionist*") OR tiab("orthodontist*") OR tiab("periodontist*") OR tiab("pharmac*") OR tiab("physical therap*") OR tiab("physician*") OR tiab("Physiotherapist*") OR tiab("social worker*")))

[S3]: [STRICT]tiab("scholarship" NEAR/3 "educational") OR tiab("scholarship" NEAR/3 "learning") OR tiab("scholarship" NEAR/3 "teaching") OR ti("scholarship")

[S4]: ([S1] OR [S2]) AND ([S3])

[S5]: [S4] AND la.exact("ENG")

ERIC (Advanced Search via EBSCO) - run July 24, 2024

S1: (DE "Medical Education" OR DE "Nursing Education" OR DE "Medical Schools" OR DE "Dental Schools" OR DE "Medical School Faculty" OR DE "Medical Students" OR DE "Nursing Students" OR DE "Allied Health Occupations Education" OR DE "Graduate Medical Education" OR DE "Pharmaceutical Education" OR TI ("health professions school*") OR AB ("health professions school*") OR TI ("Dental School*") OR AB ("Dental School*") OR TI ("dental student*") OR AB ("dental student*") OR TI ("dentistry school") OR AB ("dentistry school") OR TI ("medical college*") OR AB ("medical college*") OR TI ("medical program*") OR AB ("medical program*") OR TI ("medical school*") OR AB ("medical school*") OR TI ("medical student*") OR AB ("medical student*") OR TI ("medical teaching") OR AB ("medical teaching") OR TI ("medical training") OR AB ("medical training") OR TI ("Nursing School*") OR AB ("Nursing School*") OR TI ("nursing student*") OR AB ("nursing student*") OR TI ("pharmaceutical student*") OR AB ("pharmaceutical student*") OR TI ("pharmacy college*") OR AB ("pharmacy college*") OR TI ("Pharmacy School*") OR AB ("Pharmacy School*") OR TI ("pharmacy student*") OR AB ("pharmacy student*") OR TI ("Physiotherapy student*") OR AB ("Physiotherapy student*") OR TI ("allied health student*") OR AB ("allied health student*"))

S2: ((DE "Faculty" OR DE "Universities" OR DE "Teachers" OR DE "Colleges" OR DE "Women Faculty" OR DE "College Faculty" OR TI ("educat*") OR AB ("educat*") OR TI ("Facult*") OR AB ("Facult*") OR TI ("Pedagog*") OR AB ("Pedagog*") OR TI ("Professor*") OR AB ("Professor*") OR TI ("teaching*") OR AB ("teaching*") OR TI ("universit*") OR AB ("universit*")) AND (DE "Allied Health Occupations" OR DE "Dentistry" OR DE "Dietetics" OR DE "Nurses" OR DE "Nursing" OR DE "Physicians" OR DE "Social Work" OR DE "Allied Health Personnel" OR DE "Health Personnel" OR TI ("allied health") OR AB ("allied health") OR TI ("audiolog*") OR AB ("audiolog*") OR TI ("dental hygienist*") OR AB ("dental hygienist*") OR TI ("dental") OR TI ("dentist*") OR AB ("dentist*") OR TI ("dieti*") OR AB ("dieti*") OR TI ("endodontist*") OR AB ("endodontist*") OR TI ("health profession*") OR AB ("health profession*") OR TI ("medical") OR TI ("nurse") OR AB ("nurse") OR TI ("Nurses") OR AB ("Nurses") OR TI ("Nursing") OR TI ("nutritionist*") OR AB ("nutritionist*") OR TI ("orthodontist*") OR AB ("orthodontist*") OR TI ("periodontist*") OR AB ("periodontist*") OR TI ("pharmac*") OR AB ("pharmac*") OR TI ("physical therap*") OR AB ("physical therap*") OR TI ("physician*") OR AB ("physician*") OR TI ("Physiotherapist*") OR AB ("Physiotherapist*") OR TI ("social worker*") OR AB ("social worker*")))

S3: DE "Scholarship" OR TI ("scholarship") OR (TI(scholarship N3 educational) OR AB (scholarship N3 educational)) OR (TI (scholarship N3 learning) OR AB (scholarship N3 learning)) OR (TI (scholarship N3 teaching) OR AB (scholarship N3 teaching))

S4: (S1 OR S2) AND (S3)

S5: S4 Limits applied (Language: English)

Web of Science (Advanced Search - SCI: 1945 to 2024, AHCI: 1975 to 2024, ESCI: 2019 to 2024, SSCI: 1956 to 2024) - run July 24, 2024

#1: (TI="health professions school*" OR AB="health professions school*" OR TI="Dental School*" OR AB="Dental School*" OR TI="dental student*" OR AB="dental student*" OR TI="dentistry school" OR AB="dentistry school" OR TI="medical college*" OR AB="medical college*" OR TI="medical program*" OR AB="medical program*" OR TI="medical school*" OR AB="medical school*" OR TI="medical student*" OR AB="medical student*" OR TI="medical teaching" OR AB="medical teaching" OR TI="medical training" OR AB="medical training" OR TI="Nursing School*" OR AB="Nursing School*" OR TI="nursing student*" OR AB="nursing student*" OR TI="pharmaceutical student*" OR AB="pharmaceutical student*" OR TI="pharmacy college*" OR AB="pharmacy college*" OR TI="Pharmacy School*" OR AB="Pharmacy School*" OR TI="pharmacy student*" OR AB="pharmacy student*" OR TI="Physiotherapy student*" OR AB="Physiotherapy student*" OR TI="allied health student*" OR AB="allied health student*")

#2: ((TI="educat*" OR AB="educat*" OR TI="Facult*" OR AB="Facult*" OR TI="Pedagog*" OR AB="Pedagog*" OR TI="Professor*" OR AB="Professor*" OR TI="teaching*" OR AB="teaching*" OR TI="universit*" OR AB="universit*")

AND (TI="allied health" OR AB="allied health" OR TI="audiolog*" OR AB="audiolog*" OR TI="dental hygienist*" OR AB="dental hygienist*" OR TI="dental" OR TI="dentist*" OR AB="dentist*" OR TI="dieti*" OR AB="dieti*" OR TI="endodontist*" OR AB="endodontist*" OR TI="health profession*" OR AB="health profession*" OR TI="medical" OR TI="nurse" OR AB="nurse" OR TI="Nurses" OR AB="Nurses" OR TI="Nursing" OR TI="nutritionist*" OR AB="nutritionist*" OR TI="orthodontist*" OR AB="orthodontist*" OR TI="periodontist*" OR AB="periodontist*" OR TI="pharmac*" OR AB="pharmac*" OR TI="physical therap*" OR AB="physical therap*" OR TI="physician*" OR AB="physician*" OR TI="Physiotherapist*" OR AB="Physiotherapist*" OR TI="social worker*" OR AB="social worker*"))

#3: (TI=("scholarship" NEAR/3 "educational") OR AB=("scholarship" NEAR/3 "educational") OR TI=("scholarship" NEAR/3 "learning") OR AB=("scholarship" NEAR/3 "learning") OR TI=("scholarship" NEAR/3 "teaching") OR AB=("scholarship" NEAR/3 "teaching") OR TI="scholarship")

#4: (#1 OR #2) AND #3

#5: #4 AND English (Languages)

Scopus (Advanced Search) – run July 24, 2024

#1: ((INDEXTERMS ("allied health education" OR "allied health student" OR "dental education" OR "medical education" OR "medical school" OR "medical student" OR "nursing education" OR "nursing student" OR "pharmacy school" OR "physician assistant education" OR "dental student" OR "dental hygiene student" OR "pharmacy student")) OR (TITLE-ABS("health professions school*" OR "allied health student*" OR "Dental School*" OR "dental student*" OR "dentistry school" OR "medical college*" OR "medical program*" OR "medical school*" OR "medical student*" OR "medical teaching" OR "medical training" OR "Nursing School*" OR "nursing student*" OR "pharmaceutical student*" OR "pharmacy college*" OR "Pharmacy School*" OR "pharmacy student*" OR "Physiotherapy student*")))

#2: ((INDEXTERMS ("teaching" OR "university") OR TITLE-ABS("educat*" OR "Facult*" OR "Pedagog*" OR "Professor*" OR "teaching*" OR "universit*")) AND (INDEXTERMS ("dental hygienist" OR "dentist" OR "dietitian" OR "nurse" OR "nursing" OR "pharmacist" OR "physician" OR "physiotherapist" OR "social worker") OR TITLE("dental" OR "medical" OR "Nursing") OR TITLE-ABS("allied health" OR "allied health" OR "audiolog*" OR "dental hygienist*" OR "dentist*" OR "dieti*" OR "endodontist*" OR "health profession*" OR "nurse" OR "Nurses" OR "nutritionist*" OR "orthodontist*" OR "periodontist*" OR "pharmac*" OR "physical therap*" OR "physician*" OR "Physiotherapist*" OR "social worker*")))

#3: TITLE-ABS("scholarship" W/3 ("educational" OR "learning" OR "teaching")) OR TITLE("scholarship")

#4: (#1 OR #2) AND (#3)

#5: #4 AND (LIMIT-TO (LANGUAGE , "english"))
