# Supplementary material for: A Scoping Review of the Conceptualization, Operationalization, and Institutional Recognition of the Scholarship of Teaching and Learning in Health Professions Education: Using Institutional Logics to Understand Inconsistencies
Source: Perspect Med Educ. 2026 Jun 5;15(1):482–501. doi: 10.5334/pme.2740 (PMC13239391; doi:10.5334/pme.2740)

Supplementary Material 4

Screening Workflow

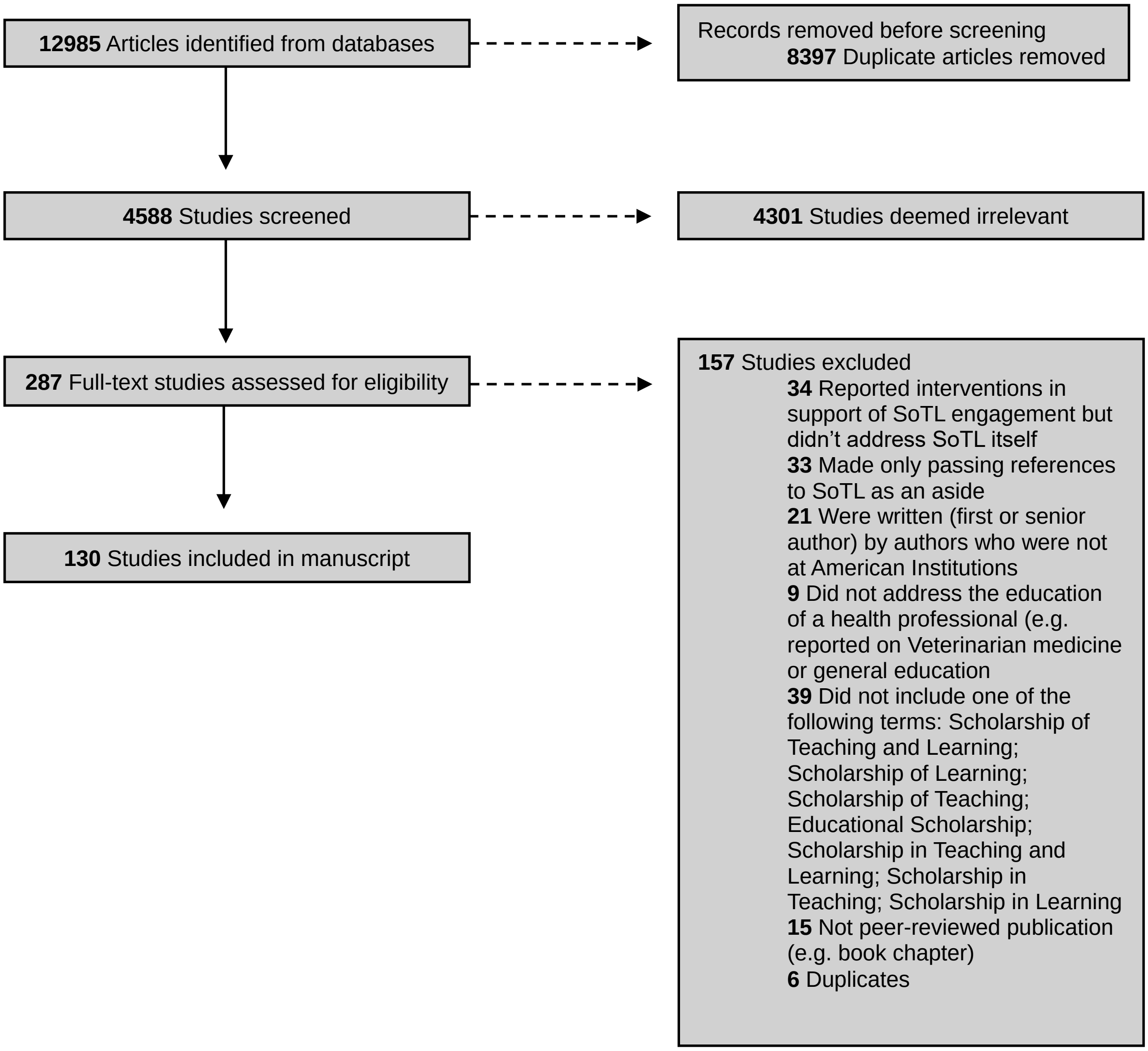

Figure 2: Publications grouped into 5-year blocks for articles in medicine and other health professions

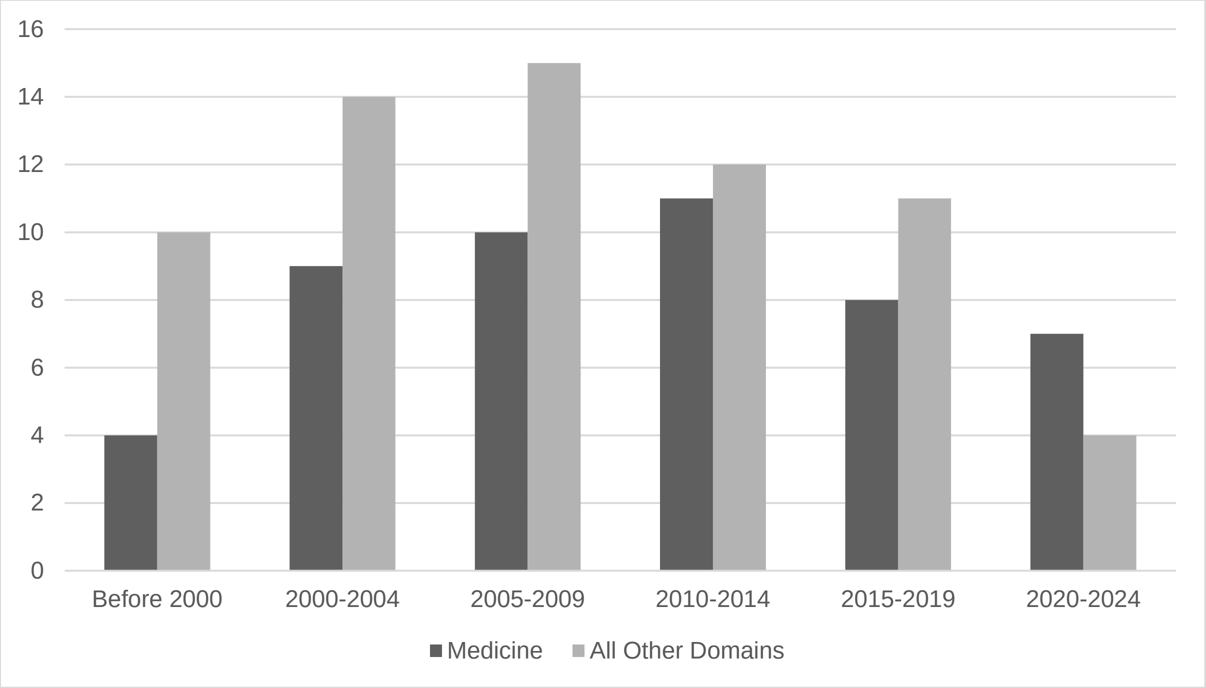

Supplement: Supplementary Material 4. — Screening Workflow. [file pme-15-1-2740-s4.pdf]
