# Supplementary material for: A Scoping Review of the Conceptualization, Operationalization, and Institutional Recognition of the Scholarship of Teaching and Learning in Health Professions Education: Using Institutional Logics to Understand Inconsistencies
Source: Perspect Med Educ. 2026 Jun 5;15(1):482–501. doi: 10.5334/pme.2740 (PMC13239391; doi:10.5334/pme.2740)
Supplement: Supplementary Material 5. — SOTL in ME: Data Extraction Tool. [file pme-15-1-2740-s5.pdf]

**Supplementary Material 5**

**SOTL in ME: Data Extraction Tool**

**Paper Demographics**

1. Paper Title: ______________________________________________________________
2. First Author: _____________________________________________________________
3. Year of Publication: ________________________________________________________
4. Who is completing this extraction: ______________________________________________
5. If data has already been extracted for this article, who completed extraction: ______________________________________________________________________

**Inclusion Decision**

6. Does the paper meet these inclusion criteria? (Select all that apply)
   - In English
   - First AND Senior (i.e. last) are from USA
   - Is a peer-reviewed journal publication
   - Manuscript addresses scholarship in the health professions education field
   - Manuscript includes some variation of and defines or develops these terms: Scholarship of teaching and learning (SoTL), Scholarship of teaching (SOT), Scholarship of learning (SOL), Scholarship in teaching and learning (SITL), Scholarship in teaching (SIT), Scholarship in learning (SIL), Educational scholarship (ES)
7. Does this manuscript meet all these inclusion criteria?
   - Yes: Continue – there are exclusion criteria to be considered too
   - No: Continue and select the exclusion criteria that are grounds for the paper's exclusion
8. Exclusion Criteria: Should the paper be rejected for any exclusion criteria (select all that apply)
   - The manuscript DOES contain reference to some variation of SOTL terms HOWEVER the term(s) is not the focus of the manuscript. In these papers, SOTL terms are only passingly referenced / not the focus of the manuscript. For example: The SOTL term(s) is present in only one or two lines of the manuscript. FOR EXAMPLE: "Studying X may lead to improved understanding of SOTL" [SOTL is a suggested area for future research] OR SOTL mentioned as part of the introduction, methodology, or

results BUT SoTL is not delved into or addressed in a meaningful way (ex. A paper that studied an implemented workshop created based on educational scholarship, where the focus of the paper is the outcome of the workshop, not educational scholarship)
  - The term SOTL terms are used as synonyms for research. The paper is actually focused on research, but not research or explanation of SOTL; instead, one of the SOTL terms is used interchangeably with research

**IF ANY EXCLUSION CRITERIA WERE SELECTED, EXIT NOW. IF NOT, CONTINUE ON.**

**SoTL in HPE Context**

9. The scope of inclusion for the literature search is the entire, broad scoped field of HPE. We want to know which professions, within that broad scope, are publishing about SoTL. Therefore, please indicate specifically which field the paper focuses on (e.g. Medicine? Nursing? Pharmacy? Physician assistant? Occupational therapy? Physical Therapy? If more than one field applies, please use "Multi"

   ________________________________________________________________________________

10. What term(s) is used in the paper?
    - Scholarship of teaching and learning (SOTL)
    - Scholarship of teaching (SOT)
    - Scholarship of learning (SOL)
    - Scholarship in teaching and learning (SOTIL)
    - Scholarship in teaching (SIT)
    - Scholarship in learning (SIL)
    - Educational scholarship (ES)
    - Other ____________________
11. How is the SOTL term defined in the paper? If a particular scholar (e.g., Boyer, OR Glassick, OR Schulman, OR…) is referred to or used in relation to the concept of the SOTL term, please describe with verbatim excerpts with page number from the manuscript

    ________________________________________________________________________________

12. Does the manuscript describe how the term has evolved or changed over time?
    - Yes
    - No
13. If YES to previous question, please describe with verbatim excerpts with page numbers from the manuscript

    ________________________________________________________________________________

14. SOTL terms are defined in abstract / theoretical ways. This allows the concept to be applied to many different contexts. However, in most papers, the abstract concept needs to be applied in more practical ways. In this question, we are

interested in learning how the abstract concept of the SOTL term is applied in the context of this manuscript. How is the SOTL term realized / brought to life / measured / framed in the paper? Please describe with verbatim excerpts with page number from the manuscript.

_______________________________________________________________________________

15. Does this paper address the promotion and/or tenure of faculty and/or clinician educators with education and/or SoTL focused careers (i.e., not research focused but teaching focused promotion pathways)?
    - Yes
    - No
16. If YES to previous question, please describe how education and/or SoTL focused careers are discussed. If possible, please use verbatim excerpts (with page numbers) from the manuscript

    _______________________________________________________________________________

17. Some papers might offer descriptions of factors that promote engagement of the SOTL term. Are any FACILITATORS noted in the paper?
    - Yes
    - No
18. If YES to previous question, please describe with verbatim excerpts with page number from the manuscript.

    _______________________________________________________________________________

19. Are any challenges noted in the manuscript?
    - Yes
    - No
20. If YES to previous question, please describe with verbatim excerpts with page number from the manuscript.

    _______________________________________________________________________________

21. Are any possible solutions to these challenges offered in the manuscript?
    - Yes
    - No
22. If YES to previous question, please describe with verbatim excerpts with page number from the manuscript.

    _______________________________________________________________________________

23. Articles found in the manuscript that should be considered for inclusion/hand search?

    _______________________________________________________________________________
