# Supplementary material for: A Scoping Review of the Conceptualization, Operationalization, and Institutional Recognition of the Scholarship of Teaching and Learning in Health Professions Education: Using Institutional Logics to Understand Inconsistencies
Source: Perspect Med Educ. 2026 Jun 5;15(1):482–501. doi: 10.5334/pme.2740 (PMC13239391; doi:10.5334/pme.2740)
Supplement: Supplementary Material 6. — Corpus Articles. [file pme-15-1-2740-s6.pdf]

Supplementary Material 6

Corpus Articles

Year	Citation
BEFORE 2000	**Medicine (N=4)**
	Jacobs MB. Faculty status for clinician-educators. *Academic Medicine*. 1993;68(2):126-128. doi:10.1097/00001888-199302000-00004
	Schneeweiss R, Ramsey PG, Jonsen AR. Reflections on the scholarship of teaching. *Academic Medicine*. 1997;72(6):481-482. doi:10.1097/00001888-199706000-00009
	Sachdeva AK, Cohen R, Dayton MT, et al. A new model for recognizing and rewarding the educational accomplishments of surgery faculty. *Academic Medicine*. 1999;74(12):1278-1287. doi:10.1097/00001888-199912000-00007
	Simpson DE, Fincher RM. Making a Case for the Teaching Scholar. *Academic Medicine*. 1999;74(12):1296-1299.
	**All Other Domains (N=10)**
	**Nursing**
	Baker C. Scholarship in Nursing: An Unpopular Subject. *The Alumnae Magazine*. 1974;70(3):14-16
	Shoffner DH, Davis MW, Bowen SM. A Model for Clinical Teaching as a Scholarly Endeavor. *Image: The Journal of Nursing Scholarship*. 1994;26(3):181-184. doi:10.1111/j.1547-5069.1994.tb00310.x
	Brown SA, Cohen SM, Kaeser L, et al. Nursing Perspective of Boyer's Scholarship Paradigm. *Nurse Educator*. 1995;20(5):26-30.
	Starck PL. Boyer's multidimensional nature of scholarship: A new framework for schools of nursing. *Journal of Professional Nursing*. 1996;12(5):268-276. doi:10.1016/S8755-7223(96)80006-8
	Bartels JE. Understanding teaching scholarship—Beyond the dichotomization. *Journal of Professional Nursing*. 1997;13(5):278. doi:10.1016/s8755-7223(97)80102-0
	Everett L, Brown S, Pokorny M, et al. Approaches to Implementing a Philosophy of Scholarship. *Nurse Educ*. 1998;23(1):13-14. doi:10.1097/00006223-199801000-00008
	Sherwen LN. When the mission is teaching: Does nursing faculty practice fit? *Journal of Professional Nursing*. 1998;14(3):137-143. doi:10.1016/S8755-7223(98)80088-4
	Wood SO, Biordi DL, Miller BA, et al. Boyer's Model of Scholarship Applied to a Career Ladder for Nontenured Nursing Faculty. *Nurse Educator*. 1998;23(3):33-40. doi:10.1097/00006223-199805000-00014
	American Association of Colleges of Nursing. Defining Scholarship for the Discipline of Nursing. *Journal of Professional Nursing*. 1999;15(6):372-376.
	**Pharmacy**
	Miller KW. Treating Teaching as a Scholarly Activity. *Am J Pharm Educ*. 1991;55:373-375.
	**HPE/MIX**
	Angstadt CN, Nieman LZ, Morahan PS. Strategies to expand the definition of scholarship for the health professions. *J Allied Health*. 1998;27(3):157-161
2000-2004	**Medicine (N= 9)**
	Beattie DS. Expanding the View of Scholarship. *Academic Medicine*. 2000;75(9):871-876.

	Glassick CE. Boyer's Expanded Definitions of Scholarship, the Standards for Assessing Scholarship, and the Elusiveness of the Scholarship of Teaching. *Academic Medicine*. 2000;75(9):877-880.
	Hafler JP, Lovejoy FH. Scholarly Activity Recorded in the Portfolios of Teacher-Clinician Faculty. *Academic Medicine*. 2000;75(6):649-652.
	Mennin SP, McGrew MC. Scholarship in teaching and best evidence medical education: synergy for teaching and learning. *Med Teach*. 2000;22(5):468-471. doi:10.1080/01421590050110722
	Simpson DE, Marcdante KW, Duthie EH, Sheehan KM, Holloway RL, Towne JB. Valuing Educational Scholarship at the Medical College of Wisconsin. *Academic Medicine*. 2000;75(9):930-934. doi:10.1097/00001888-200009000-00016
	Scott RP. Our contributions: scholarship revisited. *The Annals of Thoracic Surgery*. 2001;71(2):S30-S54. doi:10.1016/s0003-4975(00)02394-8
	Smith WL, Collins J, Lanier L, Rao VM. Redefining Scholarship in Academic Radiology. *Acad Radiol*. 2001;8(9):919-921.
	Collins J. Teacher or educational scholar? they aren't the same. *Journal of the American College of Radiology*. 2004;1(2):135-139. doi:10.1016/j.jacr.2003.11.005
	Irby DM, Hodgson CS, Muller JH. Promoting Research in Medical Education at the University of California, San Francisco, School of Medicine. *Academic Medicine*. 2004;79(10):981-984. doi:10.1097/00001888-200410000-00019
	**All Other Domains (N=14)**
	**Nursing**
	American Association of Colleges of Nursing. Position Statement on Defining Scholarship for the Discipline of Nursing. *Journal of Child and Family Nursing*. 2000;3(3):244.
	Mignor D. Who Is Going to Teach Undergraduate Clinicals? *Nursing Forum*. 2000;35(3):21-24. doi: 10.1111/j.1744-6198.2000.tb01000.x
	Weimer M. Better Scholarship "On" Teaching. *Journal of Nursing Education*. 2000;39(5):195-196.
	Witt BS, Heinrich KT. Working Smart: Turning Everyday Commitments into Scholarly Outcomes. *The Journal of Continuing Education in Nursing*. 2000;31(2):71-75. doi:10.3928/0022-0124-20000301-08
	Raff BS, Arnold J. Faculty Development: an approach to scholarship. *Nurse Educ*. 2001;26(4):159-161.
	Reece SM, Pearce CW, Melillo KD, Beaudry M. The faculty portfolio: Documenting the scholarship of teaching. *Journal of Professional Nursing*. 2001;17(4):180-186. doi:10.1053/jpnu.2001.24861
	Drevdahl DJ, Stackman RW, Purdy JM, Louie BY. Merging Reflective Inquiry and Self-Study as a Framework for Enhancing the Scholarship of Teaching. *Journal of Nursing Education*. 2002;41(9):413-419. doi:10.3928/0148-4834-20020901-10
	Pullen RL. Scholarship in practice. *Nursing Made Incredibly Easy!* 2022;20(2):47-48. doi:10.1097/01.nme.0000816528.00298.ad
	Riley JM, Beal J, Levi P, McCausland MP. Revisioning Nursing Scholarship. *Journal of Nursing Scholarship*. 2002;34(4):383-389. doi:10.1111/j.1547-5069.2002.00383.x
	Rawnsley MM. Dimensions of Scholarship and the Advancement of Nursing Science: Articulating a Vision. *Nursing Science Quarterly*. 2003;16(1):6-13. doi:10.1177/0894318402239058
	Sweitzer HF. Getting Off to a Good Start: Faculty Development in Professional Programs. *The Journal of Continuing Education in Nursing*. 2003;34(6):263-272. doi:10.3928/0022-0124-20031101-09
	Glanville I, Houde S. The scholarship of teaching: implications for nursing faculty. *Journal of Professional Nursing*. 2004;20(1):7-14. doi:10.1016/j.profnurs.2003.12.002
	**Pharmacy**

	Popovich NG, Abel SR. The need for a broadened definition of faculty scholarship and creativity. *American Journal of Pharmaceutical Education*. 2002;66(1):59-65.
	Kennedy RH, Gubbins PO, Luer M, Reddy IK, Light KE. Developing and Sustaining a Culture of Scholarship. *American Journal of Pharmaceutical Education*. 2003;67(3):1-18. doi:10.1016/s0002-9459(24)00680-6
	DiPiro J. The Scholarship of Teaching. The American Journal of Pharmaceutical Education, ed. *American Journal of Pharmaceutical Education*. 2004;68(4):1
	**Occupational Therapy**
	Haertlein C, Coppard BM, Royeen CB; Commission on Education. Scholarship and occupational therapy (2003 concept paper). *Am J Occup Ther*. 2003;57(6):641-643. doi:10.5014/ajot.57.6.641
2005-2009	**Medicine (N=10)**
	Cottrell S. A matter of explanation: assessment, scholarship of teaching and their disconnect with theoretical development. *Medical Teacher*. 2006;28(4):305-308. doi:10.1080/01421590600627581
	E Fincher RM, Work JA. Perspectives on the scholarship of teaching. *Medical Education*. 2006;40(4):293-295. doi: 10.1111/j.1365-2929.2006.02404.x
	Klingensmith ME, Anderson KD. Educational scholarship as a route to academic promotion: a depiction of surgical education scholars. *The American Journal of Surgery*. 2006;191(4):533-537. doi:10.1016/j.amjsurg.2006.01.007
	Wood BP, May W. Academic Recognition of Educational Scholarship. *Academic Radiology*. 2006;13(2):254-257. doi:10.1016/j.acra.2005.10.001
	Simpson D, Fincher RME, Hafler JP, et al. Advancing educators and education by defining the components and evidence associated with educational scholarship. *Medical Education*. 2007;41(10):1002-1009. doi: 10.1111/j.1365-2923.2007.02844.x
	Christiaanse ME, Russell EL, Crandall SJ, Lambros A, Manuel JC, Kirk JK. Development of an asset map of medical education research activity. *Journal of Continuing Education in the Health Professions*. 2008;28(3):186-193. doi:doi.org/10.1002/chp.173
	Morahan PS, Fleetwood J. The double helix of activity and scholarship: building a medical education career with limited resources. *Med Educ*. 2007;42(1):34-44. doi:10.1111/j.1365-2923.2007.02976.x
	Schrader C, Barsan WG, Gordon JA, et al. Scholarship in Emergency Medicine in an Environment of Increasing Clinical Demand: Proceedings from the 2007 Association of American Medical Colleges Annual Meeting. *Academic Emergency Medicine*. 2008;15(6):567-572. doi:10.1111/j.1553-2712.2008.00118.x
	McGaghie WC. Scholarship, publication, and career advancement in health professions education: AMEE Guide No. 43. *Med Teach*. 2009;31(7):574-590. doi:10.1080/01421590903050366
	Ruiz JG, Candler CS, Qadri SS, Roos BA. E-Learning as Evidence of Educational Scholarship: A Survey of Chairs of Promotion and Tenure Committees at U.S. Medical Schools. *Academic Medicine*. 2009;84(1):47-57. doi:10.1097/ACM.0b013e3181901004
	**All Other Domains (N=15)**
	**Nursing**
	Smith MJ, Liehr P. Story Theory. *Holistic Nursing Practice*. 2005;19(6):272-276. doi:10.1097/00004650-200511000-00008
	Stull A, Lantz C. An Innovative Model for Nursing Scholarship. *Journal of Nursing Education*. 2005;44(11):493-497. doi:10.3928/01484834-20051101-04
	Bartels JE. Preparing Nursing Faculty for Baccalaureate-Level and Graduate-Level Nursing Programs: Role Preparation for the Academy. *Journal of Nursing Education*. 2007;46(4):154-158. doi:10.3928/01484834-20070401-03
	Becker KL, Dang D, Jordan E, et al. An evaluation framework for faculty practice. *Nursing Outlook*. 2007;55(1):44-54.

	doi:10.1016/j.outlook.2006.10.001
	Eddy LL. Evaluation Research as Academic Scholarship. *Nurs Educ Perspect*. 2007;28(2):77-81.
	Spath ML. A Need for Clarity: Scholarship, Scholarly Teaching, and the Scholarship of Teaching and Learning. *Nurs Educ Perspect*. 2007;28(5).
	**Physical Therapy**
	Haddad A, Jensen G. Scholarship of Teaching and Learning in Physical Therapist Education. *Journal of Physical Therapy Education*. 2005;19(3):3-4. doi:10.1097/00001416-200510000-00002
	Hinman MR, Darden A. Beyond Scholarly Teaching: Opportunities for Engaging in the Scholarship of Teaching and Learning in Physical Therapist Education. *Journal of Physical Therapy Education*. 2005;19(3):14-22. doi: 10.1097/00001416-200510000-00004
	Musolino GM, Mostrom E. Reflection and the Scholarship of Teaching, Learning, and Assessment. *Journal of Physical Therapy Education*. 2005;19(3):52-66. doi:10.1097/00001416-200510000-00008
	Peterson CA, Sandholtz JH. New faculty development: scholarship of teaching and learning opportunities. *Journal of Physical Therapy Education*. 2005;19(3):23-29.
	Spake E, Salem A. Setting the Stage: The Scholarship of Teaching and Learning. *Journal of Physical Therapy Education*. 2005;19(3):5-13.
	Wise HH, Brotherton SS, Mitcham MD. From scholarly teaching to the scholarship of teaching: a retrospective look at strategies that foster the transition of scholarly work in allied health into credible forms of scholarship. *J Allied Health*. 2008;37(1):e50-68.
	**HPE/Mix**
	Fidler DC, Khakoo R, Miller LA. Teaching Scholars Programs: Faculty Development for Educators in the Health Professions. *Academic Psychiatry*. 2007;31(6):472-478. doi:10.1176/appi.ap.31.6.472
	Smesny AL, Williams JS, Brazeau GA, Weber RJ, Matthews HW, Das SK. Barriers to Scholarship in Dentistry, Medicine, Nursing, and Pharmacy Practice Faculty. *Am J Pharm Educ*. 2007;71(5):91. doi:10.5688/aj710591
	**Occupational Therapy**
	American Occupational Therapy Association (AOTA). Scholarship in Occupational Therapy. *American Journal of Occupational Therapy*. 2009;63(6):790-796.
2010-2014	**Medicine (N = 11)**
	Geraci SA, Hollander H, Babbott SF, et al. AAIM Report on Master Teachers and Clinician Educators Part 4: Faculty Role and Scholarship. 2010;123(11):1065-1069. doi:10.1016/j.amjmed.2010.07.005
	Greenberg L, Bickel J. Teaching Scholarship and the Clinician/educator. *Pediatr Ann*. 2010;39(2):106-110. doi:10.3928/00904481-20100120-11
	LaMantia J, Kuhn GJ, Searle NS. The CORD Academy for Scholarship in Education in Emergency Medicine. *Academic Emergency Medicine*. 2010;17(s2). doi:10.1111/j.1553-2712.2010.00895.x
	Grigsby RK, Thorndyke L. Perspective: Recognizing and Rewarding Clinical Scholarship. *Academic Medicine*. 2011;86(1):127-131. doi:10.1097/ACM.0b013e3181ffae5e
	Nuthalapaty FS, Casey PM, Cullimore AJ, et al. To the point: a primer on medical education research. *American Journal of Obstetrics and Gynecology*. 2012;207(1):9-13. doi:10.1016/j.ajog.2011.12.025
	Searle NS, Teal CR, Richards BF, et al. A Standards-Based, Peer-Reviewed Teaching Award to Enhance a Medical School's Teaching Environment and Inform the Promotions Process. *Academic Medicine*. 2012;87(7):870-876. doi:10.1097/ACM.0b013e3182584130

	Shah BJ, Rose S. Scholarship in Education: The Currency for Career Development for Clinician–Educators in Gastroenterology and Hepatology. *Gastroenterology*. 2012;142(4):684-689. doi:10.1053/j.gastro.2012.02.031
	Turner T, Palazzi D, Ward M, Lorin M. Transforming teaching into scholarship. *The Clinical Teacher*. 2012;9(6):363-367. doi:10.1111/j.1743-498x.2012.00597.x
	Yarris LM, Coates WC. Creating educational leaders: experiences with two education fellowships in emergency medicine. *Acad Emerg Med*. 2012;19(12):1481-1485. doi:10.1111/acem.12042
	Yarris LM, Coates WC, Lin M, et al. A suggested core content for education scholarship fellowships in emergency medicine. *Acad Emerg Med*. 2012;19(12):1425-1433. doi:10.1111/acem.12032
	Crites GE, Gaines JK, Cottrell S, et al. medical education scholarship: An introductory guide: AMEE Guide No. 89. *Med Teach*. 2014;36(8):657-674. doi:10.3109/0142159X.2014.916791
	**All Other Domains (N=12)**
	**Nursing**
	Robert RR, Pape TM. Scholarship in nursing: not an isolated concept. *PubMed*. 2011;20(1):41-44.
	Silva MC. The Scholarship of Teaching as Science and as Art. *Journal of Nursing Education*. 2012;51(11):599-601. doi:10.3928/01484834-20121023-01
	Slimmer L. A teaching mentorship program to facilitate excellence in teaching and learning. *J Prof Nurs*. 2012;28(3):182-185. doi:10.1016/j.profnurs.2011.11.006
	McNeal GJ. Academic scholarship redefined. *ABNF J*. 2014;25(1):3-4.
	Oermann MH. Defining and Assessing the Scholarship of Teaching in Nursing. *Journal of Professional Nursing*. 2014;30(5):370-375. doi:10.1016/j.profnurs.2014.03.001
	**Pharmacy**
	Andurkar S, Fjortoft N, Sincak C, Todd T. Development of a Center for Teaching Excellence. *American Journal of Pharmaceutical Education*. 2010;74(7):123. doi:10.5688/aj7407123
	Medina M, Hammer D, Rose R, et al. Demonstrating excellence in pharmacy teaching through scholarship. *Currents in Pharmacy Teaching and Learning*. 2011;3(4):255-259. doi:10.1016/j.cptl.2011.07.008
	Poirier T, Behnen E. Where and how to search for evidence in the education literature: the WHEEL. *Am J Pharm Educ*. 2014;78(4):70. doi:10.5688/ajpe78470
	**Physical Therapy**
	Anderson JR, Tunney NM. The Scholarship of Teaching and Learning in a Physical Therapy Program. *New Directions for Teaching and Learning*. 2014;2014(139):61-76. doi:10.1002/tl.20105
	Spake EF. Perspectives on pediatric physical therapy education. *Pediatr Phys Ther*. 2014;26(1):2-6. doi:10.1097/PEP.0000000000000002
	**HPE/Mix**
	Burns S, Merchant C, Appelt E. Campus Survey on the Status of the Scholarship of Teaching and Learning (SOTL) by Health Sciences Faculty. *Education*. 2013;133(4):502-512.
	**Occupational Therapy**
	Gupta J, Bilics A. Scholarship and research in occupational therapy education. *Am J Occup Ther*. 2014;68 Suppl 2:S87-S92. doi:10.5014/ajot.2014.012880
	**Dentistry**

	Jahangiri L, Mucciolo TW. Toward a model of institutional scholarship in health professions education. *J Dent Educ*. 2011;75(12):1569-1576.
	Karimbux NY. Promoting the scholarship of teaching and learning through peer review and public presentation. *J Dent Educ*. 2014;78(10):1351-1352.
	Lanning SK, McGregor M, Crain G, Van Ness CJ, Keselyak NT, Killip JW. The status of the scholarship of teaching and learning in dental education. *J Dent Educ*. 2014;78(10):1353-1363.
2015-2019	**Medicine (N=8)**
	Linaker KL. Radiologists as Educators: A Narrative Review of the Literature. *J Chiropr Humanit*. 2015;22(1):22-26. Published 2015 Nov 16. doi:10.1016/j.echu.2015.09.003
	Jordan J, Jones D, Williams D, Druck J. Publishing Venues for Education Scholarship: A Needs Assessment. *Academic Emergency Medicine*. 2016;23(6):731-735. doi:10.1111/acem.13003
	Ander D, Love J. The Evolving Definition of Education Scholarship: What the Clinician Educator Needs to Know. *Western Journal of Emergency Medicine*. 2017;18(1):1-3. doi:10.5811/westjem.2016.12.33326
	Clarke SO, Jordan J, Yarris LM, et al. The View From the Top: Academic Emergency Department Chairs' Perspectives on Education Scholarship. Blanchard RD, ed. *AEM Education and Training*. 2017;2(1):26-32. doi:10.1002/aet2.10070
	Darden AG, DeLeon SD. Transitioning From Medical Educator to Scholarship in Medical Education. *Am J Med Sci*. 2017;353(2):137-144. doi:10.1016/j.amjms.2016.11.015
	Kyle BN, Corral I, John NJ, Shelton PG. Educational Scholarship and Technology: Resources for a Changing Undergraduate Medical Education Curriculum. *Psychiatric Quarterly*. 2017;88(2):249-261. doi:10.1007/s11126-016-9474-7
	Franzen D, Cooney R, Chan T, Brown M, Diercks DB. Scholarship by the Clinician-Educator in Emergency Medicine. *AEM Educ Train*. 2018;2(2):115-120. doi:10.1002/aet2.10084
	Irby DM, O'Sullivan PS. Developing and rewarding teachers as educators and scholars: remarkable progress and daunting challenges. *Med Educ*. 2018;52(1):58-67. doi:10.1111/medu.13379
	O'Brien BC, Irby DM, Durning SJ, et al. Boyer and Beyond: An Interview Study of Health Professions Education Scholarship Units in the United States and a Synthetic Framework for Scholarship at the Unit Level. *Acad Med*. 2019;94(6):893-901. doi:10.1097/ACM.0000000000002625
	**All Other Domains (N=11)**
	**Nursing**
	Nosek CM, Scheckel MM, Waterbury T, MacDonald A, Wozney N. The Collaborative Improvement Model: An Interpretive Study of Revising a Curriculum. *J Prof Nurs*. 2017;33(1):38-50. doi:10.1016/j.profnurs.2016.05.006
	Oermann MH. Building Your Scholarship From Your Teaching: Plan Now. *Nurse Educ*. 2017;42(5):217. doi:10.1097/NNE.0000000000000417
	Opacic DA, Roessler E. Defining Scholarship in Physician Assistant Education. *Journal of Physician Assistant Education*. 2017;28(3):143-145. doi:10.1097/JPA.0000000000000136
	American Association of Colleges of Nursing. Defining Scholarship for Academic Nursing. *Journal of Professional Nursing*. 2018;34(3):149-156. doi:10.1016/j.profnurs.2018.04.004
	Hartjes TM. Academic nursing scholarship redefined. *J Am Assoc Nurse Pract*. 2018;30(12):664-666. doi:10.1097/JXX.0000000000000156
	Minnick A, Kleinpell R, Norman LD. Promoting Faculty Scholarship: A Clinical Faculty Scholars Program. *J Nurs Educ*.

	2018;57(2):121-125. doi:10.3928/01484834-20180123-11
	**Pharmacy**
	Bosso JA, Hastings JK, Speedie MK, Rodriguez de Bittner M. Recommendations for the successful pursuit of scholarship by pharmacy practice faculty members. *Am J Pharm Educ*. 2015;79(1):04. doi:10.5688/ajpe79104
	Mehvar R. Why Every Aspect of an Academic Pharmacy Career Should Be Viewed Through the Lens of Scholarship. *Am J Pharm Educ*. 2017;81(1):2. doi:10.5688/ajpe8112
	Mospan CM. Management education within pharmacy curricula: A need for innovation. *Curr Pharm Teach Learn*. 2017;9(2):171-174. doi:10.1016/j.cptl.2016.11.019
	**HPE/Mix**
	Register SJ, King KM. Promotion and Tenure: Application of Scholarship of Teaching and Learning, and Scholarship of Engagement Criteria to Health Professions Education. *Health Professions Education*. 2018;4(1):39-47. doi:10.1016/j.hpe.2017.02.002
	**Occupational Therapy**
	Hammel J, Magasi S, Mirza MP, et al. A Scholarship of Practice Revisited: Creating Community-Engaged Occupational Therapy Practitioners, Educators, and Scholars. *Occup Ther Health Care*. 2015;29(4):352-369. doi:10.3109/07380577.2015.1051690
2020-2026	**Medicine (N= 7)**
	Hoffman LA, Lufler RS, Brown KM, et al. A review of U.S. Medical schools' promotion standards for educational excellence. *Teach Learn Med*. 2020;32(2):184-193. doi:10.1080/10401334.2019.1686983
	Jacobs CK, Everard KM, Cronholm PF. Promotion of Clinical Educators: A Critical Need in Academic Family Medicine. *Fam Med*. 2020;52(9):631-634. doi:10.22454/FamMed.2020.687091
	Blanco M, Prunuske J, DiCorcia M, Learman LA, Mutcheson B, Huang GC. The DoCTRINE Guidelines: Defined Criteria to Report Innovations in Education. *Academic Medicine*. 2022;97(5):689-695. doi:10.1097/ACM.0000000000004634
	Beck Dallaghan GL, Wright ST, Plant J, Butani L, Morgenstern BZ. Educational Relative Value Units as a Measure of Academic Productivity: A Systematic Review. *Cureus*. Published online April 1, 2023. doi:10.7759/cureus.36995
	Milner RJ, Flotte TR, Thorndyke LE. Defining Scholarship for Today and Tomorrow. *Journal of Continuing Education in the Health Professions*. 2023;43(2):133-138. doi:10.1097/CEH.0000000000000473
	Bockrath R, Osman C, Trainor J, et al. Education Scholarship Assessment Reconsidered: Expansion of Glassick's Criteria to Incorporate Health Equity. *Academic Medicine*. 2024;99(5):487-492. doi:10.1097/ACM.0000000000005654
	Cochran A, Stefanidis D, Kibbe MR. Improving the Integrity of Surgical Education Scholarship. *JAMA Surg*. 2024;159(3):237-238. doi:10.1001/jamasurg.2023.6672
	Gribble ML, Gunther JR, Braunstein SE. Radiation Oncology Education Across the Learning Continuum: Current Challenges and Future Directions. *Semin Radiat Oncol*. 2026;37:150999. doi:10.1016/j.semradonc.2026.150999
	Parlapalli RS, O'Dorisio N, Frank MG, Merchant NB. Pathways to promotion: Making everyday work count towards scholarship opportunities. *J Hosp Med*. 2026;21(2):210-214. doi:10.1002/jhm.70177
	**All Other Domains (N=4)**
	**Nursing**
	Howard PB, Williams TE, El-Mallakh P, et al. An innovative teaching model in an academic-practice partnership for a Doctor of Nursing Practice program. *J Prof Nurs*. 2020;36(5):285-291. doi:10.1016/j.profnurs.2020.04.010
	Clark R, Stanfill AG. Lessons Learned From Pairing Education-Intensive and Research-Intensive Faculty to Increase Scholarship in Nursing. *Nurs Educ Perspect*. 2021;42(5):323-324. doi:10.1097/01.NEP.0000000000000822

	Matthias AD, Scott MD, Ivins T, Osinski J. Enhancing the Relevance and Use of Information Literacy in Future Nurse Educators. *Nurs Educ Perspect*. 2021;42(5):327-328. doi:10.1097/01.NEP.0000000000000848
	Butcher HK. Disciplinary Thinking for the Scholarship of Nursing Research. *Nursing Science Quarterly*. 2024;38(1):8-17. doi:10.1177/08943184241291548
	**Pharmacy**
	Franks AM, Payakachat N. Positioning the Scholarship of Teaching and Learning Squarely on the Center of the Desk. *American Journal of Pharmaceutical Education*. 2020;84(9):1169-1174. doi:10.5688/ajpe8046
	Islam MA, Taheri R, McBane S, Talukder R. Faculty assessment of scholarship of teaching and learning among United States pharmacy programs. *Currents in Pharmacy Teaching and Learning*. 2020;12(2020). doi:10.1016/j.cptl.2020.04.025
	Kennedy DR, Beckett RD, O'Donnell LA. Strategies, Ideas, and Lessons Learned While Engaging in SoTL Without Formal Training. *Am J Pharm Educ*. 2020;84(1):7702. doi:10.5688/ajpe7702
	Dy-Boarman EA, Wippermann B, Janke KK. Scholarly maturation and other factors supporting publishing productivity in educational scholarship for clinical faculty. *Currents in Pharmacy Teaching and Learning*. 2021;13(11):1398-1407. doi:10.1016/j.cptl.2021.09.019
	Forrest J, Elnaem MH, Gleason SE, Birnie C, Ryan M. White paper on the scholarship of teaching and learning: Expanding the academic pharmacy promotion and tenure process. *Pharmacy Education*. 2022;22(4). doi:10.46542/pe.2022.224.115122
	**Occupational Therapy**
	American Occupational Therapy Association (AOTA). Scholarship in Occupational Therapy. *American Journal of Occupational Therapy*. 2022;76(Supplement_3):1-6. doi:10.5014/ajot.2022.76s3011
**Total Medicine Articles: 52**	
**Total Other Domains Articles: 78**	
**Total Articles in Corpus: 130**	
