# Supplementary material for: A Scoping Review of the Conceptualization, Operationalization, and Institutional Recognition of the Scholarship of Teaching and Learning in Health Professions Education: Using Institutional Logics to Understand Inconsistencies
Source: Perspect Med Educ. 2026 Jun 5;15(1):482–501. doi: 10.5334/pme.2740 (PMC13239391; doi:10.5334/pme.2740)
Supplement: Supplementary Material 7. — Explicit and Implicit Reference to Seminal SOTL Scholars by Article. [file pme-15-1-2740-s7.pdf]

Supplementary Material 7

Explicit and Implicit Reference to Seminal SOTL Scholars by Article

	Boyer (1990)		Glassick et al (1997)		Hutchings & Shulman (1999)		No Definition	Defined but not according to Boyer, Glassick et al, or Hutchings & Shulman
	Explicit Reference	Implicit Reference	Explicit Reference	Implicit Reference	Explicit Reference	Implicit Reference		
**MEDICINE**								
Jacobs 1993	x							
Schneeweiss 1997	x							
Sachdeva 1999	x							
Simpson 1999	x							
Beattie 2000	x							
Glassick 2000	x				x			
Hafler 2000							x	
Mennin 2000	x		x		x			
Simpson 2000	x							
Scott 2001	x							
Smith 2001	x							
Collins 2004	x		x		x			
Irby 2004	x							
Cottrell 2006	x			x	x			
Fincher 2006	x				x			
Klingensmith 2006							x	
Wood 2006	x				x			
Morahan 2007	x		x		x			
Simpson 2007	x							
Christiaanse 2008	x		x					
Schrader 2008	x							
McGaghie 2009	x		x					
Ruiz 2009					x			
Geraci 2010	x			x				
Greenberg 2010	x							
Lamantia 2010	x					x		
Grigsby 2011	x		x					
Nuthalapaty 2012	x							
Searle 2012	x							
Shah 2012	x		x			x		
Turner 2012	x		x					
Yarris 2012	x		x					
Yarris 2012							x	
Crites 2014			x					
Linaker 2015							x	
Jordan 2016							x	
Ander 2017	x		x		x			

Darden 2017	x		x					
Kyle 2017						x		
Clarke 2018								x
Franzen 2018								x
Irby 2018	x		x					
O’Brien 2019	x							
Hoffman 2020	x		x					
Jacobs 2020	x							
Blanco 2022								x
Beck/Dallaghan 2023	x		x					
Milner 2023	x		x					
Bockrath 2024	x		x					
Cochran 2024	x		x					
Gribble 2026					x			
Parlapalli 2026	x							
**Medicine Totals**	**40**	**0**	**18**	**2**	**10**	**3**	**5**	**3**
**ALL OTHER DOMAINS**								
**Nursing Articles**								
Baker 1974								x
Shoffner 1994	x							
Brown 1995	x							
Starck 1996	x							
Bartels 1997		x						
Everett 1998	x							
Sherwen 1998	x							
Wood 1998	x							
AACN 1999	x							
AACN 2000	x							
Mignor 2000	x							
Weimer 2000	x							
Witt 2000	x							
Raff 2001	x							
Reece 2001	x				x			
Drevdahl 2002							x	
Pullen 2002	x							
Riley 2002	x				x			
Rawnsley 2003	x							
Sweitzer 2003		x			x			
Glanville 2004	x							
Smith 2005	x		x					
Stull 2005	x							
Bartels 2007	x	x						
Becker 2007	x							
Eddy 2007	x							
Spath 2007				x		x		
Robert 2011	x							
Silva 2012						x		
Slimmer 2012							x	
McNeal 2014	x							
Oermann 2014							x	
Nosek 2017	x				x			

Oermann 2017				x				
Opacic 2017	x			x				
AACN 2018	x							
Hartjes 2018		x						
Minnick 2018							x	
Howard 2020	x							
Clark 2021							x	
Matthias 2021							x	
Butcher 2025	x		x					
**Total**	**29**	**4**	**2**	**3**	**4**	**2**	**6**	**1**
**Pharmacy**								
Miller 1991	x							
Popovich 2002	x				x			
Kennedy 2003	x		x		x			
DiPiro 2004				x				
Andurkar 2010							x	
Medina 2011				x		x		
Poirier 2014							x	
Bosso 2015							x	
Mehvar 2017				x				
Mospan 2017							x	
Franks 2020	x				x			
Islam 2020	x					x		
Kennedy 2020				x				
Dy-Boarman 2021								x
Forrest 2022	x							
**Total**	**6**	**0**	**1**	**4**	**3**	**2**	**4**	**1**
**Physical Therapy**								
Haddad 2005							x	
Hinman 2005	x		x		x			
Musolino 2005	x		x		x			
Peterson 2005	x				x			
Spake 2005	x		x		x			
Wise 2008	x		x		x			
Anderson 2014								x
Spake 2014					x			
**Total**	**5**	**0**	**4**	**0**	**6**	**0**	**1**	**1**
**HPE/Mix**								
Angstadt 1998	x							
Fidler 2007							x	
Smesny 2007	x							
Burns 2013				x		x		
Register 2018	x				x			
**Total**	**3**	**0**	**0**	**1**	**1**	**1**	**1**	**0**
**Occupational Therapy**								
Haertlein 2003	x							
AOTA 2009	x			x				

Gupta 2014	x					x		
Hammel 2015							x	
AOTA 2022	x			x				
**Total**	**4**	**0**	**0**	**2**	**0**	**1**	**1**	**0**
**Dentistry**								
Jahangiri 2011	x		x					
Karimbux 2014				x				
Lanning 2014			x					
**Total**	**1**	**0**	**2**	**1**	**0**	**0**	**0**	**0**
**All Other Domains Totals:**	**48**	**4**	**9**	**11**	**14**	**6**	**13**	**3**
**Totals in Corpus**	**88**	**4**	**27**	**13**	**24**	**9**	**18**	**6**
