# Supplementary material for: A Scoping Review of the Conceptualization, Operationalization, and Institutional Recognition of the Scholarship of Teaching and Learning in Health Professions Education: Using Institutional Logics to Understand Inconsistencies
Source: Perspect Med Educ. 2026 Jun 5;15(1):482–501. doi: 10.5334/pme.2740 (PMC13239391; doi:10.5334/pme.2740)
Supplement: Supplementary Material 8. — SOTL Operationalizations by Audience Dissemination Focus. [file pme-15-1-2740-s8.pdf]

Supplementary Material 8

SOTL Operationalizations by Audience Dissemination Focus

	Public Dissemination				Local Dissemination				Public or Local Dissemination			
	Peer-reviewed Publications	Presentations	Books	Grant Funding	Teaching	Clinical Practice	Teaching Portfolios	Evidence Based Learning	New Courses / Curricula	Peer Recognition	Creating Educational Resources	Assessment / Evaluation
**MEDICINE**												
Jacobs 1993					x		x		x	x	x	
Schneeweiss 1997												
Sachdeva 1999					x				x		x	
Simpson 1999							x					
Beattie 2000	x											
Glassick 2000												
Hafler 2000												
Mennin 2000												
Simpson 2000												
Scott 2001												
Smith 2001											x	x
Collins 2004							x		x	x		
Irby 2004												
Cottrell 2006												
Fincher 2006	x										x	x
Klingensmith 2006												
Wood 2006							x					
Simpson 2007												
Christiaanse 2008	x	x										
Morahan 2008												
Schrader 2008											x	
McGaghie 2009					x							
Ruiz 2009					x							
Geraci 2010					x	x	x		x	x	x	x
Greenberg 2010												
Lamantia 2010								x				
Grigsby 2011	x											
Nuthalapaty 2012												
Searle 2012							x					
Shah 2012	x											x
Turner 2012	x											
Yarris 2012							x					
Crites 2014												
Linaker 2015												
Jordan 2016	x								x		x	x
Ander 2017					x				x			x
Darden 2017												

Kyle 2017											x	x
Clarke 2018	x				x				x			
Franzen 2018					x		x		x			x
Irby 2018												
O'Brien 2019												
Hoffman 2020												
Jacobs 2020									x			
Blanco 2022												
Beck Dallaghan 2023	x	x		x								
Milner 2023	x		x						x		x	x
Bockrath 2024												
Cochran 2024												
Gribble 2026	x											x
Parlapalli 2026	x	x	x		x	x			x		x	x
**Medicine Totals**	**12**	**3**	**2**	**1**	**9**	**2**	**8**	**1**	**11**	**3**	**10**	**11**
**ALL OTHER DOMAINS**												
**Nursing Articles**												
Baker 1974												
Shoffner 1994												
Brown 1995									x			
Starck 1996									x			x
Bartels 1997					x							
Everett 1998												
Sherwen 1998			x									
Wood 1998	x	x		x								
AACN 1999	x	x		x	x				x	x	x	x
AACN 2000					x				x	x	x	x
Mignor 2000					x						x	
Weimer 2000												
Witt 2000												
Raff 2001												
Reece 2001	x	x	x	x	x		x		x	x	x	
Drevdahl 2002												
Pullen 2002												
Riley 2002							x					
Rawnsley 2003												
Sweitzer 2003												
Glanville 2004												
Smith 2005												
Stull 2005					x			x	x			x
Bartels 2007			x		x				x		x	x
Becker 2007						x						
Eddy 2007									x			x
Spath 2007												
Robert 2011	x			x	x				x	x	x	x
Silva 2012												
Slimmer 2012	x	x		x	x							x
McNeal 2014	x			x								x
Oermann 2014	x				x						x	x
Nosek 2017												
Oermann 2017	x								x		x	x

Opacic 2017												
AACN 2018	x		x	x	x			x	x		x	x
Hartjes 2018	x							x	x		x	x
Minnick 2018												
Howard 2020												
Clark 2021												
Matthias 2021												
Butcher 2025					x							
**Nursing Totals**	**10**	**4**	**4**	**7**	**12**	**1**	**2**	**3**	**12**	**4**	**10**	**13**
**Pharmacy**												
Miller 1991	x		x									
Popovich 2002		x			x				x		x	x
Kennedy 2003	x	x			x				x		x	
DiPiro 2004	x											
Andurkar 2010											x	
Medina 2011	x	x			x						x	x
Poirier 2014												
Bosso 2015												
Mehvar 2017	x											x
Mospan 2017												
Franks 2020	x	x			x							x
Islam 2020	x	x	x	x	x		x		x		x	x
Kennedy 2020												
Dy-Boarman 2021	x			x	x				x	x		
Forrest 2022	x	x	x	x	x				x		x	x
**Total**	**9**	**6**	**3**	**3**	**7**	**0**	**1**	**0**	**5**	**1**	**6**	**6**
**Physical Therapy**												
Haddad 2005												
Hinman 2005									x			x
Musolino 2005							x					
Peterson 2005												
Spake 2005					x							
Wise 2008												
Anderson 2014								x				
Spake 2014												
**Total**	**0**	**0**	**0**	**0**	**1**	**0**	**1**	**1**	**1**	**0**	**0**	**1**
**HPE/Mix**												
Angstadt 1998					x	x			x	x	x	x
Fidler 2007												
Smesny 2007	x	x										
Burns 2013	x	x		x							x	
Register 2018	x		x	x	x		x		x	x	x	x
**Total**	**3**	**2**	**1**	**2**	**2**	**1**	**1**	**0**	**2**	**2**	**3**	**2**
**Occupational Therapy**												
Haertlein 2003												x
AOTA 2009						x						
Gupta 2014	x											

Hammel 2015											x	
AOTA 2022					x			x				
**Total**	**1**	**0**	**0**	**0**	**1**	**1**	**0**	**1**	**0**	**0**	**1**	**1**
**Dentistry**												
Jahangiri 2011												
Karimbux 2014	x	x										
Lanning 2014	x	x			x						x	
**Total**	**2**	**2**	**0**	**0**	**1**	**0**	**0**	**0**	**0**	**0**	**1**	**0**
**All Other Domains Total:**	**25**	**14**	**8**	**12**	**24**	**3**	**5**	**5**	**20**	**7**	**21**	**23**
**Totals in Corpus**	**37**	**17**	**10**	**13**	**33**	**5**	**13**	**6**	**31**	**10**	**31**	**34**
